# Lumpy skin disease virus 001/156 protein is a virulence factor that suppresses interferon production through impairing IRF3 dimerization

**DOI:** 10.1371/journal.ppat.1013362

**Published:** 2025-07-23

**Authors:** Minmin Zhang, Yujie Shi, Xinyin Lu, Qiwei Zhang, Yubo Zhao, Shaohan Li, Zhiyuan Wen, Jinying Ge, Xijun Wang, Jie Li, Zhigao Bu, Xin Yin

**Affiliations:** 1 State Key Laboratory for Animal Disease Control and Prevention, Harbin Veterinary Research Institute, Chinese Academy of Agricultural Sciences, Harbin, China; 2 Jinyu Biotechnology Co., LTD, Hohhot, China; University of California, School of Medicine, UNITED STATES OF AMERICA

## Abstract

Lumpy skin disease virus (LSDV), a member of the genus *Capripoxvirus* within the family *Poxviridae*, causes significant disease in cattle and is classified as a notifiable disease by the World Organization for Animal Health (WOAH). The virus contains a double-stranded linear DNA genome of approximately 151 kbp, encoding 156 predicted open reading frames (ORFs) for various proteins. However, only a limited number of these proteins have been characterized, with the functions of many—particularly those encoded within the inverted terminal repeat (ITR) regions—remaining largely unknown. In this study, we utilized homologous recombination to generate LSDV mutants with deletions of the LSDV 001/156 gene to investigate its role. LSDV 001/156, an uncharacterized protein located within the ITR region, was identified as a late-expressed gene product incorporated into virions and involved in viral replication. Further analysis revealed that LSDV 001/156 acts as a negative regulator of the interferon (IFN) signaling pathway. It interacts with interferon regulatory factor 3 (IRF3), disrupting its dimerization and nuclear translocation, thereby attenuating IFN production. Functional studies demonstrated that the LSDV mutant lacking the 001/156 gene exhibited reduced replication and virulence in cattle compared to the wild-type virus, likely due to enhanced IFN responses in the absence of this immune-evasive protein. In summary, our findings uncover a novel role of the LSDV 001/156 gene in modulating the host intrinsic antiviral response, shedding light on the mechanisms underlying LSDV pathogenesis. This study highlights the importance of ITR-encoded genes in immune evasion and virulence, providing new insights into LSDV biology and its interactions with the host immune system.

## Author summary

The inverted terminal repeat (ITR) regions in certain poxviruses have been previously characterized; however, the gene composition and functional roles of ITR regions in lumpy skin disease virus (LSDV) remain unclear. LSDV 001/156, located in the ITR region of LSDV and present in two copies within the viral genome, encodes a late gene product involved in viral replication. This protein impedes dimerization and nuclear translocation of interferon regulatory factor 3 (IRF3), thereby suppressing type I interferon (IFN-I) production. Importantly, LSDV 001/156 contributes to virulence *in vivo*, revealing a novel mechanism by which LSDV evades the host innate immune response. These findings enrich our understanding of viral pathogenesis and immune evasion strategies.

## Introduction

The variola virus, the first identified poxvirus in humans, caused smallpox—a devastating disease that was eradicated in 1980 through extensive vaccination efforts, despite limited understanding of the virus at the time. However, other members of the *Poxviridae* family continue to circulate in nature, posing ongoing threats to both human and animal health. In early 2022, as the COVID-19 pandemic held global focus, an international mpox outbreak unfolded, starting with clusters in Europe. Traced to a strain circulating in Nigeria since 2017, the outbreak revealed escalating public health challenges as the virus spread beyond its endemic regions. On 14 August 2024, the World Health Organization (WHO) declared mpox a Public Health Emergency of International Concern (PHEIC) for the second time [1]. The expansion of international trade and travel has facilitated the widespread transmission of poxvirus-related diseases, adversely affecting livestock health and global food security. Although substantial progress has been made in understanding poxvirus transmission and pathogenicity, our knowledge of these viruses remains incomplete, underscoring the critical need for continued research and sustained vigilance to mitigate their impact on public and animal health.

The poxvirus genome is organized into three distinct regions: the conserved central region, variable regions, and inverted terminal repeat sequences (ITRs) located at each end [2]. The central region contains highly conserved essential genes responsible for critical viral functions, including entry, transcription, replication, and assembly [3–6]. In contrast, the variable regions near the ends of the genome contain genes associated with host range and virulence, facilitating adaptation to specific hosts, potentially by inhibiting inflammatory and antiviral signaling pathways [7–9]. ITRs are inverted repeats consisting of identical DNA sequences arranged in opposing orientations at each end of the genome. This structural symmetry enables the formation of hairpin or cruciform secondary structures that play essential roles in initiating replication and stabilizing the poxviral genome [10]. The length of ITRs varies significantly across poxviruses, and gene duplication within these regions contributes to their adaptation to new environments [11–13]. Despite their importance, the functions of proteins encoded within ITRs remain poorly understood. This knowledge gap poses a significant challenge to the development of effective control measures and interventions against poxvirus-associated diseases.

The innate immune response serves as the body’s first line of defense against pathogens, initiating when pattern recognition receptors (PRRs) detect conserved pathogen-associated molecular patterns (PAMPs) [14–16]. Key PRRs, such as RIG-I and cGAS, recognize viral RNA and DNA, respectively, triggering robust antiviral responses [17,18]. A central component of PRR signaling is interferon regulatory factor 3 (IRF3), which plays an essential role in the transcription of interferons (IFNs) [19]. Upon PAMP detection, IRF3 undergoes phosphorylation, enabling its translocation to the nucleus with the help of importins. In the nucleus, IRF3 binds to promoter regions of type I interferon (IFN-I) genes, inducing their expression. The secreted IFNs act on nearby cells, activating them to produce a range of antiviral proteins encoded by IFN-stimulated genes (ISGs). Together, this cascade ultimately promotes viral clearance and bolsters the innate immune response [20,21].

In contrast, viruses have evolved sophisticated mechanisms to evade, modulate, and manipulate host innate immune responses [22,23]. The *Poxviridae* family offers a valuable model for studying these countermeasures. For instance, vaccinia virus (VACV) encodes the E3L protein, which binds double-stranded RNA (dsRNA) to inhibit protein kinase R (PKR) activation, a key component of the IFN-mediated antiviral response [24]. Similarly, ectromelia virus produces EVM166, an IFN-I decoy receptor, while the myxoma virus expresses M062 to potentially inhibit DNA-sensing-dependent IFN responses [25,26]. VACV B2 targets cyclic GMP-AMP (cGAMP), thereby disrupting the production of type I IFNs downstream of cGAS-STING pathway activation [27]. Additionally, poxvirus proteins with conserved B-cell lymphoma (Bcl-2)-like structural folds, such as B14, N1, A52, C6, A46, and K7 modulate immune responses by targeting distinct components of the IFN and NF-κB signaling pathways [28–33]. These examples demonstrate the diverse strategies employed by poxviruses to suppress host antiviral responses. While many immunomodulators are encoded within the variable regions of the viral genome, the roles of genes located in the ITR regions remain underexplored. Further investigation into ITR genes is crucial for uncovering novel mechanisms of poxvirus immune evasion and pathogenicity.

Lumpy skin disease virus (LSDV) is a member of the genus *Capripoxvirus* within the family *Poxviridae*. It is the causative agent of lumpy skin disease (LSD), a highly contagious viral disease that primarily affects cattle, leading to significant economic losses in the livestock industry [34]. Due to its potential to cause widespread outbreaks, LSD is classified as a notifiable disease by the World Organization for Animal Health (WOAH). LSD was first identified in Zambia in 1929 and has subsequently spread across Africa, the Middle East, Europe, and parts of Asia, primarily due to international trade and livestock movement. Transmission occurs primarily through biting insects such as *Stomoxys* spp. (stable flies) and *Culicoides* spp. (biting midges), as well as through direct contact with infected animals or contaminated materials [34,35]. The disease poses a major threat to food security and livestock health worldwide, urgently necessitating the development of effective control measures.

LSDV has a double-stranded linear DNA genome approximately 151 kilobase pairs (kbp) in size, with an AT content of about 73%. The genome is organized into a conserved central region, responsible for essential functions such as viral replication, and variable terminal regions, including ITRs at both ends [36]. The ITRs are approximately 2.4 kb in length and encode four open reading frames (ORFs), yet the specific roles of these genes in the viral life cycle remain poorly understood.

In this study, we characterized the LSDV 001/156 gene, which is duplicated within the ITR regions of the LSDV genome. LSDV 001/156 is incorporated into virions [37], is expressed late during infection, and plays a crucial role in viral replication. We showed that LSDV 001/156 inhibits the IFN-I signaling pathway by blocking IRF3 dimerization. *In vivo* studies demonstrated that deletion of LSDV 001/156 significantly attenuated the virulence of the LSDV Xinjiang/2019 strain in cattle, highlighting its role in immune evasion. In conclusion, our findings uncover a novel mechanism by which LSDV 001/156 downregulates type I IFN production, thereby enhancing LSDV virulence and providing critical insights into the pathogenesis of LSDV.

## Results

### LSDV 001/156 significantly affects LSDV replication but is not indispensable

**Fig 1:**
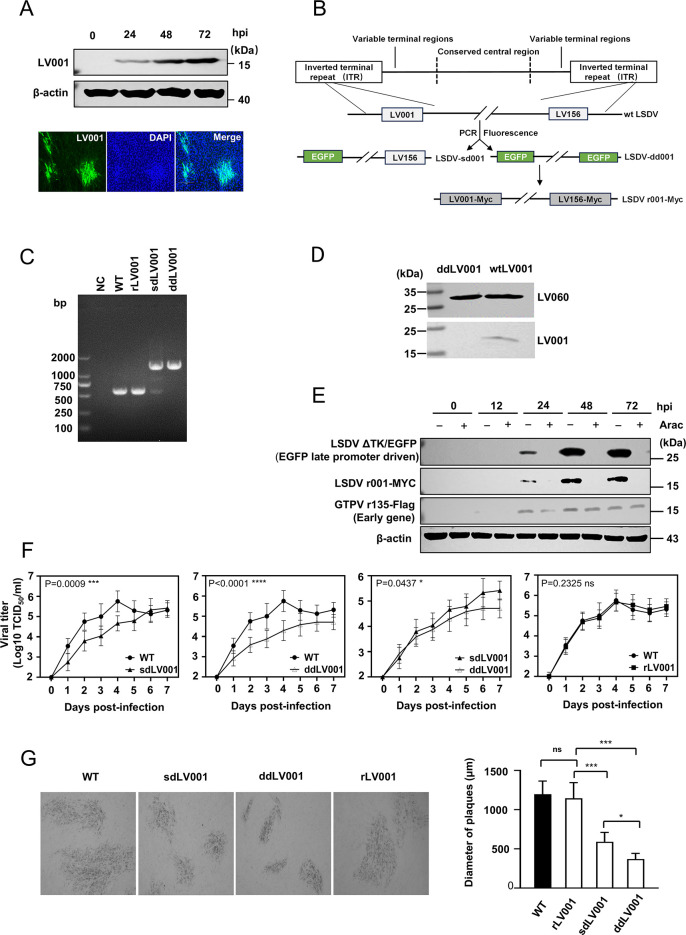
LV001 is a late-expressed gene that influences LSDV replication *in vitro.* (A) Western blotting analysis confirmed the expression of the LV001/156 protein (approximately 18 kDa). (B) Schematic representation of the genomes of the wild-type (WT) and recombinant LSDV/China/Xinjiang/2019 strains. (C) Various recombinant viruses are identified by PCR and sequencing. (D) LT cells were infected with LSDV-WT or LSDV-dd001/156. Viral particles were subsequently purified by sucrose cushion ultracentrifugation protocol. The presence of LV001 in purified virions was assessed by Western blotting. (E) LV001 was identified as a late-expressed gene. LT cells were infected with LSDV ΔTK/EGFP, LSDV r001/156-Myc, and GTPV r135-FLAG at a multiplicity of infection (MOI) of 5 in the presence or absence of AraC. Expression levels of LV001/156, EGFP, GTPV 135, and β-actin were detected by Western blotting. (F) Although the LV001 gene is not essential for LSDV replication, it significantly affects viral titers. LT cells were infected with LSDV-WT, LSDV r001/156-Myc, LSDV-sd001/156, or LSDV-dd001/156 at an MOI of 0.05. Cells and supernatants were harvested at different time points by freeze-thawing three times, followed by low-speed centrifugation. Supernatants were collected for viral titration in LT cells. Data represent independent experiments performed in triplicate. Statistical significance was determined using two-way ANOVA using repeated measures (*P < 0.05, **P < 0.01, ***P < 0.001, ****P < 0.0001). (G) Comparison of plaque formation by different recombinant viruses. LT cells were infected with LSDV-WT, LSDV r001/156-Myc, LSDV-sd001/156, or LSDV-dd001/156 at an MOI of 0.5, and cytopathic effects were observed at 96 h post-infection. The diameters of 15 plaques were measured. Statistical significance was determined using an unpaired Student’s *t*-test (*P < 0.05, **P < 0.01, ***P < 0.001).

LSDV 001/156 (referred to as LV001), located within the ITRs of LSDV genome, encodes proteins with previously uncharacterized functions. To confirm its expression during infection, we analyzed the expression of LV001 in primary lamb testicle (LT) cells infected with the LSDV Xinjiang/2019 strain (LSDV-WT). Western blotting analysis using a polyclonal antibody specific to LV001 detected an 18 kDa band, which was additionally verified by indirect immunofluorescence staining (Fig 1A). To determine its role in LSDV virulence *in vitro*, we generated two mutants, LSDV-sd001/156 (single-gene deletion) and LSDV-dd001/156 (double-gene deletion), via homologous recombination using the Xinjiang/2019 strain as the backbone. In these mutants, both LV001 and LV156 were replaced by an enhanced green fluorescent protein (EGFP) gene cassette under the control of the p11 promoter. Additionally, to confirm the phenotypic effects of LV001 deletion, a revertant mutant (LSDV r001/156-Myc) was constructed using the LSDV-dd001/156 strain as the template with LV001 reinserted into the viral genome under its native promoter. In this revertant, the LV001 open reading frame (ORF) was restored with a C-terminal Myc tag, replacing the EGFP cassette (Fig 1B). The nucleotide sequences of both parental and revertant viruses in the 5’ region were shown in S1 Fig. Recombinant viruses were screened and purified using a plaque assay (S2A Fig). The successful construction of all mutants was validated through Western blotting, PCR and sequencing analyses (Figs 1C, S2B, and S2C). These recombinant strains provided essential tools to explore the specific functions of LV001 in viral replication and virulence.

LV001 has previously been identified as a component of viral particles [37]. Consistently, the LV001 protein was detected in the virus particles purified by sucrose density gradient centrifugation (Fig 1D). To determine whether LV001 functions expressed as an early or late gene during LSDV infection, LT cells were infected with the recombinant viruses LSDV r001/156-Myc, LSDV ΔTK/EGFP, and GTPV r135-FLAG at a multiplicity of infection (MOI) of 0.1. The infections were performed both in the presence and absence of arabinofuranosyl cytosine (AraC), a known inhibitor of DNA replication and late gene expression. The analysis revealed that LV001 expression was completely inhibited by AraC, similar to the late protein thymidine kinase (TK), as indicated by the absence of EGFP expression in LSDV ΔTK/EGFP-infected cells. In contrast, the early gene 135 from GTPV was expressed regardless of the presence of AraC, consistent with previous findings [38]. These results confirmed that LV001 is a late gene, dependent on viral DNA replication for its expression (Fig 1E). To evaluate the effect of LV001 on viral replication, we compared the growth kinetics of the parental LSDV strain and recombinant mutants in LT cells. Both single-gene deletion mutant (LSDV-sd001/156) and double-gene deletion mutant (LSDV-dd001/156) exhibited significantly reduced viral yields, approximately 5- to 10-fold lower than those of the parental LSDV and the revertant strain LSDV r001/156-Myc (Fig 1F). Additionally, plaque morphology analysis revealed differences in the plaque sizes produced by the recombinant viruses. The plaque size was significantly reduced in cells infected with LSDV-sd001/156 and LSDV-dd001/156, whereas cells infected with LSDV-WT and the revertant variant exhibited large plaques (Fig 1G). These findings suggest that unlike most variable region genes, which are typically expressed early during infection, LV001 is a late gene that significantly influences viral replication.

### LV001 inhibited IFN-β production in infected cells

**Fig 2:**
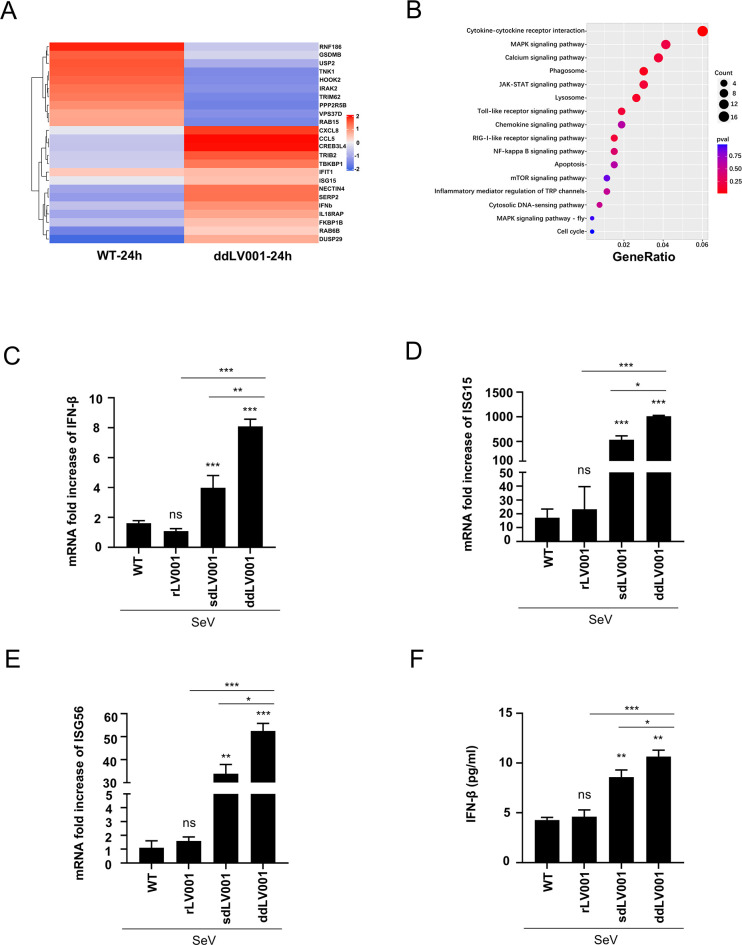
The absence of LV001 promoted IFN-β production in infected cells. (A) Heatmap showing differentially expressed genes (DEGs) involved in the IFN response induced by LSDV-dd001/156 and LSDV-WT. Transcriptome sequencing (RNA-seq) analysis was performed on MDBK cells infected with LSDV-dd001/156 or LSDV-WT at an MOI of 2, and total RNA was extracted at the indicated time points. The total mRNA from MDBK cells was used to generate RNA-seq libraries. (B) Kyoto Encyclopedia of Genes and Genomes (KEGG) pathway enrichment analysis of LSDV-dd001/156- and WT LSDV-infected MDBK cells. (C − E) LV001/156 inhibits the production of type I interferons (IFN-I) and interferon-stimulated genes (ISGs). MDBK cells were infected with LSDV-dd001/156, LSDV-sd001/156, the revertant mutant LSDV r01-156-Myc, or LSDV-WT at an MOI of 5. At 20 h post-infection (hpt), the cells were stimulated with Sendai virus (SeV) for 16 h, and the mRNA levels of IFN-β (C), ISG15 (D), and ISG56 (E) were quantified using reverse transcription-quantitative PCR (RT-qPCR). (F) LSDV-dd001/156 promotes IFN-β production. MDBK cells were infected with LSDV-dd001/156, LSDV-sd01/156, revertant mutant LSDV r01/156-Myc, or LSDV-WT at an MOI of 3 for 24 h, followed by SeV stimulation for 16 h. The protein level of bovine IFN-β (bo-IFN-β) was then measured by ELISA. Error bars represent standard errors of the mean. Data were analyzed using Student’s *t*-test: *P < 0.05; **P < 0.01.

The infectivity of poxviruses relies heavily on their ability to modulate host antiviral responses [7]. Since the deletion of LV001 impairs LSDV replication *in vitro*, and given that the MDBK cells, derived from bovine tissue, support robust LSDV replication, we conducted a comprehensive analysis of differentially expressed genes (DEGs) in MDBK cells infected with either LSDV-dd001/156 or LSDV-WT at an MOI of 2. Transcriptome sequencing (RNA-seq) revealed that genes associated with the IFN signaling pathway were significantly upregulated in LSDV-dd001/156-infected cells compared to LSDV-WT-infected cells at 24 hours post-infection (hpi) (Fig 2A). To further explore the pathways affected by LV001, we performed Kyoto Encyclopedia of Genes and Genomes (KEGG) pathway enrichment analysis. The analysis indicated that the upregulated genes were enriched in pathways related to IFN signaling, including cytosolic DNA-sensing, RIG-I-like receptor signaling, Toll-like receptor signaling, and JAK-STAT pathways (Fig 2B). Given that many known poxvirus proteins with Bcl-2-like folds exhibit similar functions in antagonizing IFN signaling activation, we performed a detailed amino acid sequence alignment analysis comparing LV001 with its orthologs from the *Orthopoxvirus* and *Capripoxvirus* genera (S3 Fig). The analysis revealed that key structural motifs are conserved across these orthologs. Specifically, LV001 shares 35% sequence identity with its homologs in vaccinia virus and monkeypox virus, respectively, while exhibiting 100% identity with homologs within the same genus. To confirm LV001’s role in regulating the IFN signaling pathway, we measured the transcriptional levels of IFN-β and ISGs using reverse transcription quantitative polymerase chain reaction (RT-qPCR). MDBK cells were infected with LSDV-sd001/156, LSDV-dd001/156, LSDV r001/156-Myc, or LSDV-WT at an MOI of 5, followed by stimulation with Sendai virus (SeV). Results showed that LSDV-dd001/156 infection significantly increased the transcription of IFN-β compared to LSDV-WT and the revertant mutant (Fig 2C). Further analysis revealed that LSDV-dd001/156 infection also enhanced the expression of downstream ISGs, including ISG15 and ISG56, particularly in response to SeV stimulation (Fig 2D and 2E). In parallel, MDBK cells were infected with the same virus panel (MOI = 5) in the presence or absence of AraC, without SeV stimulation. Consistent with previous results, LSDV-dd001/156 infection led to elevated IFN-β and ISG transcription (S4 Fig). These findings were corroborated by enzyme-linked immunosorbent assays (ELISAs), which showed increased levels of IFN-β in the supernatants of mutant-infected LT cells following SeV stimulation (Fig 2F). These results collectively demonstrate that LV001 functions as a suppressor of the IFN signaling pathway, aiding the virus in evading the host’s innate immune response.

### LV001 inhibited IFN-β signal transduction

**Fig 3:**
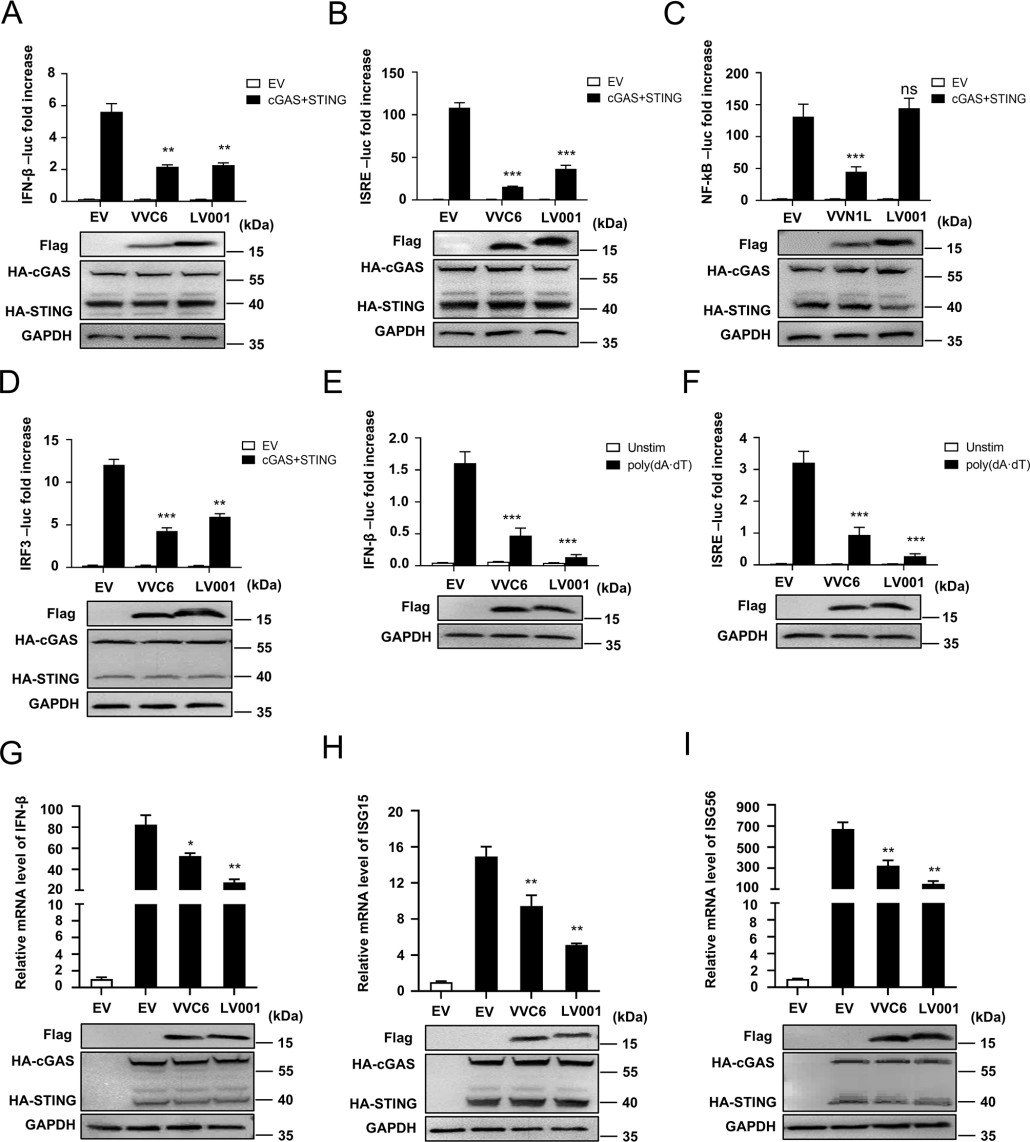
LV001 inhibits IFN-βsignal transduction. (A-D) HEK293T cells were transfected with the firefly luciferase (Luc) reporter gene under the control of (A) IFN-β, (B) ISRE, (C) NF-κB, or (D) the IRF3 promoter, along with the pRL-TK plasmid. LV001, VACV C6 (VV C6), and VV N1L, were included as experimental conditions, with an empty vector (EV) serving as negative control. HEK293T cells were cultured in 48-well plates and transfected with 100 ng/well of the firefly luciferase reporter plasmid, 10 ng/well of pRL-TK, or 100 ng/well of the cGAS-HA or STING-HA expression plasmid. At 24 h post-transfection (hpt), promoter activation was measured using a dual-luciferase assay kit. (E, F) HEK293T cells were transfected with 100 ng/well of IFN-β-Luc or ISRE-Luc plasmids and 10 ng/well of pRL-TK. After 24 hpt, the cells were stimulated with poly(dA·dT) (2 mg/mL) for 12 h. The activation of the IFN-β and ISRE promoters was evaluated via a dual-luciferase assay. (G − I) HEK293T cells were transfected with the FLAG-LV001 expression plasmid along with the HA-cGAS and HA-STING plasmids. At 24 hpt, the mRNA levels of IFN-β, ISG15, and ISG56 were quantified by qPCR. Data were presented as mean ± SD and represent one of at least three independent experiments carried out in triplicate. Statistical significance was determined using an unpaired Student’s *t*-test: *P < 0.05; **P < 0.01 compared to EV.

Cytosolic DNA sensors such as cGAS and RNA polymerase III (RNA Pol III) play pivotal roles in triggering IFN-I responses to Poxvirus infections [39,40]. To investigate the role of LV001 in IFN-I signal transduction, HEK293T cells were co-transfected with a firefly luciferase reporter plasmid, either a FLAG empty vector or FLAG-LV001, and plasmids expressing cGAS-HA and STING-HA. As positive controls, VACV C6 and N1L were included, known for inhibiting IRF3/IRF7 phosphorylation and NF-κB signaling, respectively [30,41]. At 24 hours post-transfection (hpt), luciferase activity was measured to evaluate promoter activation. The results demonstrated that LV001 overexpression significantly suppressed cGAS/STING-induced activation of the IFN-β, interferon-stimulated response element (ISRE), and IRF3 promoters (Fig 3A–3D). Notably, LV001 did not affect the activation of the NF-κB promoter, suggesting a specific mechanism of inhibition targeting the IRF3 pathway. Moreover, LV001 inhibited the activation of IFN-β and ISRE promoters in response to poly(dA·dT) stimulation, a synthetic analog of cytosolic double-stranded DNA (dsDNA) that mimics viral DNA (Fig 3E and 3F). To confirm the inhibitory role of LV001 at the transcriptional level, the expression of IFN-β and ISGs was analyzed in HEK293T cells. Cells expressing LV001 exhibited significantly reduced mRNA levels of IFN-β and ISGs, including ISG15 and ISG56, following cGAS/STING pathway activation (Fig 3G–3I). Collectively, these findings demonstrate that LV001 inhibits cGAS/STING- and poly(dA·dT)-induced IFN-β production.

### LV001 inhibits the IFN-β signaling pathway by targeting IRF3

**Fig 4:**
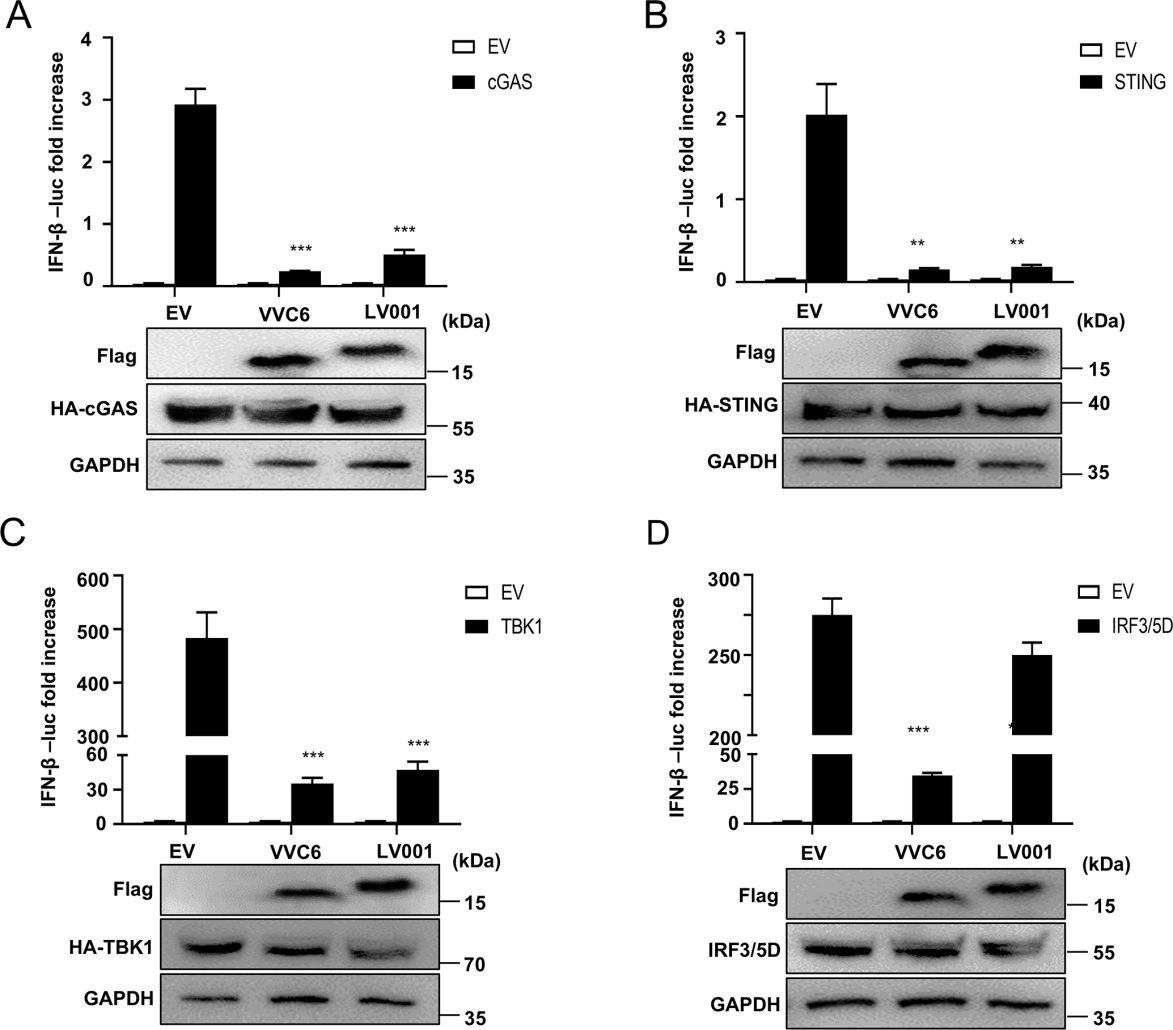
LV001 inhibits the IFN-βsignaling pathway by targeting IRF3. (A − D) HEK293T cells were cotransfected with 100 ng/well of IFN-β or ISRE luciferase (Luc) reporter plasmid, 10 ng/well of the pTK-Rluc plasmid expressing Renilla luciferase, and expression plasmids for cGAS-HA (A), STING (B), TBK1 (C), or IRF3-5D, the activated form of IRF3 (D), along with either a FLAG-LV001 expression plasmid or an empty vector. Luciferase activity was measured 24 h post-transfection (hpt). The expression levels of cGAS, STING, TBK1, IRF3-5D, and LV001 were assessed by Western blotting using mouse anti-HA or anti-FLAG monoclonal antibodies, with GAPDH serving as loading control. The error bars represent the standard errors of the means. Statistical significance was determined using Student’s *t*-test: *P < 0.05, **P < 0.01.

**Fig 5:**
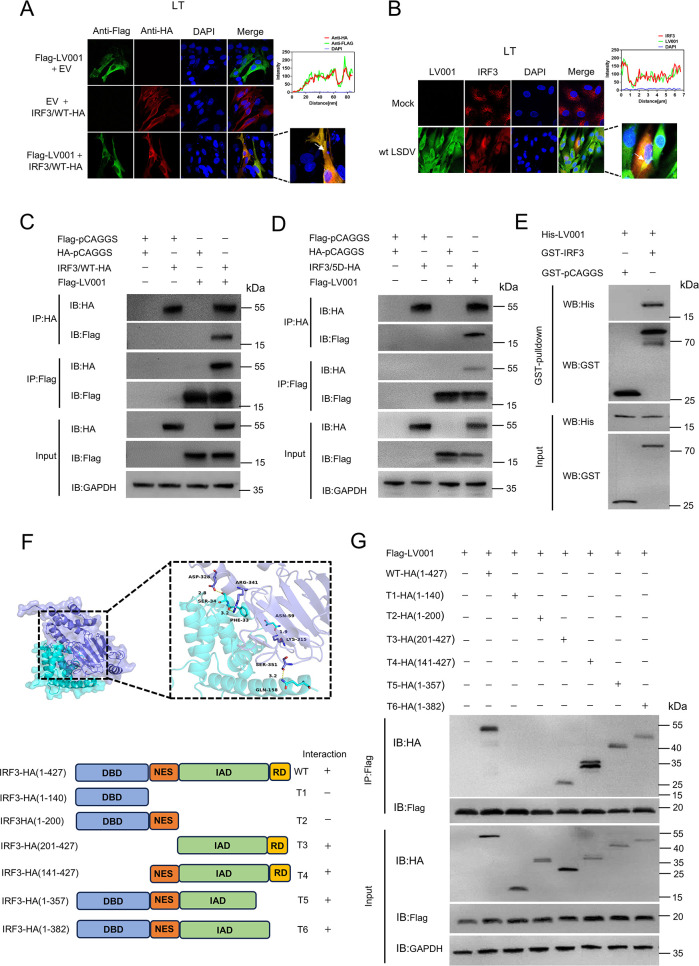
LV001 directly interacts with IRF3. (A, B) LT cells were either transfected with plasmids encoding FLAG-LV001 and IRF3-HA or infected with wild-type LSDV. After fixation, cells were stained with anti-FLAG and anti-HA antibodies, or with antibodies against LV001 and polyclonal antibodies against IRF3. The subcellular localization of LV001 and IRF3 was visualized by confocal microscopy. Fluorescence signals corresponding to FLAG-LV001 and IRF3-HA (A), or endogenous LV001 and IRF3 (B), were analyzed using ImageJ. (C, D) HEK293T cells were transfected with plasmids expressing HA-IRF3 and FLAG-LV001, followed by immunoprecipitation at 24 h post-transfection (hpt) with anti-FLAG or anti-HA antibodies. The whole-cell lysates and immunoprecipitates were analyzed by Western blotting. (E) A GST pulldown assay was performed in which the purified glutathione S-transferase (GST) fusion protein IRF3 (GST-IRF3) or GST alone was incubated with purified His-LV001. (F) AlphaFold2 was used to predict the three-dimensional model of the interaction between IRF3 and LV001, along with a schematic representation of full-length IRF3 and its truncated mutants. (G) HEK293T cells were cotransfected with plasmids that express FLAG-LV001 and IRF3-HA or its truncated mutants. At 24 hpt, the cell lysates were immunoprecipitated with an anti-FLAG antibody, and the whole-cell lysates and immunoprecipitates were analyzed by Western blotting.

**Fig 6:**
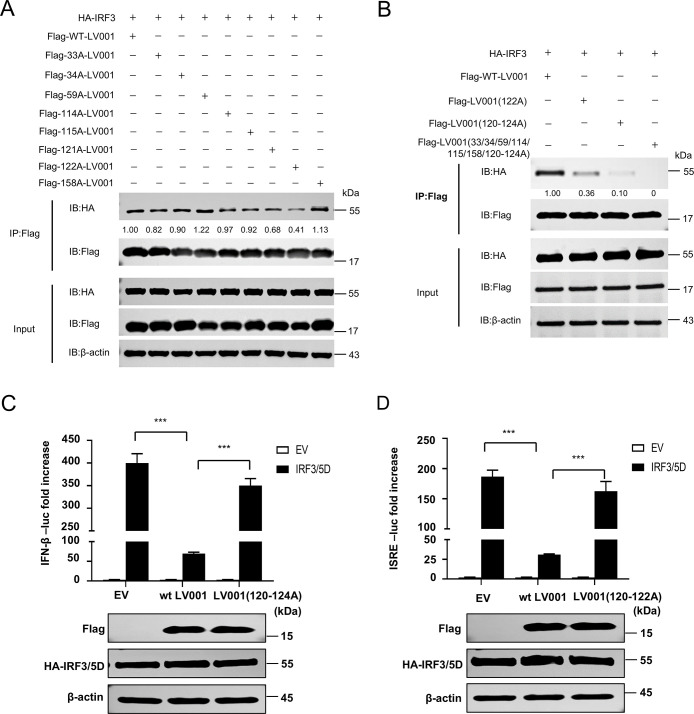
LV001 Requires IRF3 Binding to Inhibit IFN-I Production. (A-B) HEK293T cells were transfected with plasmids expressing HA-IRF3 and a series of FLAG-tagged LV001 mutants, followed by immunoprecipitation at 24 h post-transfection (hpt) with anti-FLAG or anti-HA antibodies. The whole-cell lysates and immunoprecipitates were analyzed by Western blotting. (C-D) HEK293T cells were cultured in 48-well plates and transfected with 100 ng/well of the firefly luciferase reporter plasmid, 10 ng/well of pRL-TK, and 100 ng/well of the IRF3/5D-HA and FLAG-LV001(122A) or Flag-LV001(120-124A) expression plasmid. At 24 h post-transfection (hpt), promoter activation was measured using a dual-luciferase assay kit. The expression levels of IRF3, LV001, and LV001(120-124A) were assessed by Western blotting using mouse anti-HA or anti-FLAG monoclonal antibodies, with β-actin serving as loading control. The error bars represented the standard errors of the means. Statistical significance was determined using Student’s t-test: *P < 0.05, **P < 0.01, ***P < 0.001.

To identify the specific target of LV001 in the IFN signaling pathway, HEK293T cells were co-transfected with pFLAG-LV001 and luciferase reporter plasmids, along with expression constructs for cGAS-HA, STING-HA, TBK1-HA, or IRF3/5D-HA (an constitutively active form of IRF3) [19]. LV001 significantly suppressed the activation of the IFN-β promoter induced by all these proteins (Fig 4), indicating that LV001 acts at the level of IRF3 or downstream in the signaling pathway. To determine the correlation between pFLAG-LV001 and pIRF3-HA, co-transfection or LSDV-WT infection were performed in LT cells. Confocal microscopy revealed that LV001 and IRF3 colocalized in the cytoplasm, with Manders’ colocalization coefficients of 0.95 and 0.91, respectively (Fig 5A and 5B). A co-immunoprecipitation (Co-IP) assay further validated this interaction, as LV001 co-precipitated with IRF3 (Fig 5C and 5D). Additionally, a glutathione S-transferase (GST) pulldown assay confirmed that the recombinant LV001 protein, but not GST alone, directly interacted with IRF3 (Fig 5E). Consistently, surface plasmon resonance (SPR) analysis confirmed a strong direct interaction between these two proteins, with a dissociation constant (K_D_) of 5.20 × 10 ⁻ ⁸ M (S5 Fig). IRF3 consists of four distinct domains: an N-terminal DNA-binding domain (DBD), nuclear export sequence (NES), IRF association domain (IAD), and a C-terminal regulatory domain (RD) [42]. Using AlphaFold2, the 3D structure of the LV001-IRF3 interaction was predicted, revealing that LV001 primarily binds to residues 315–341 of IRF3 (Fig 5F). To determine which domains of IRF3 interacted with LV001, an additional Co-IP assay was performed, which revealed that the deletion of the DBD, NES, or RD of IRF3 did not affect its interaction with LV001, suggesting that IRF3 interacts with LV001 through IAD (Fig 5F and 5G). Based on the predicted interaction interface, we generated a series of LV001 mutants. The LV001(120–124A) mutant showed significantly weakened IRF3 binding in Co-IP assays (Fig 6A and 6B) and substantially impaired IFN suppression activity in dual-luciferase reporter assays (Fig 6C and 6D). Additionally, we generated a combinatorial mutant in which all predicted IRF3-interacting residues were simultaneously substituted. This extensively mutated construct completely lost its ability to bind IRF3 (Fig 6B). While this result may reflect disruption of key interface residues, we cannot exclude the possibility that the extensive mutations affected the overall protein stability or folding. Collectively, these findings demonstrate that LV001 inhibits IFN-β signaling by directly targeting IRF3 through its IAD domain.

### LV001 inhibits the IFN-β production signaling pathway by disrupting the dimerization of IRF3

**Fig 7:**
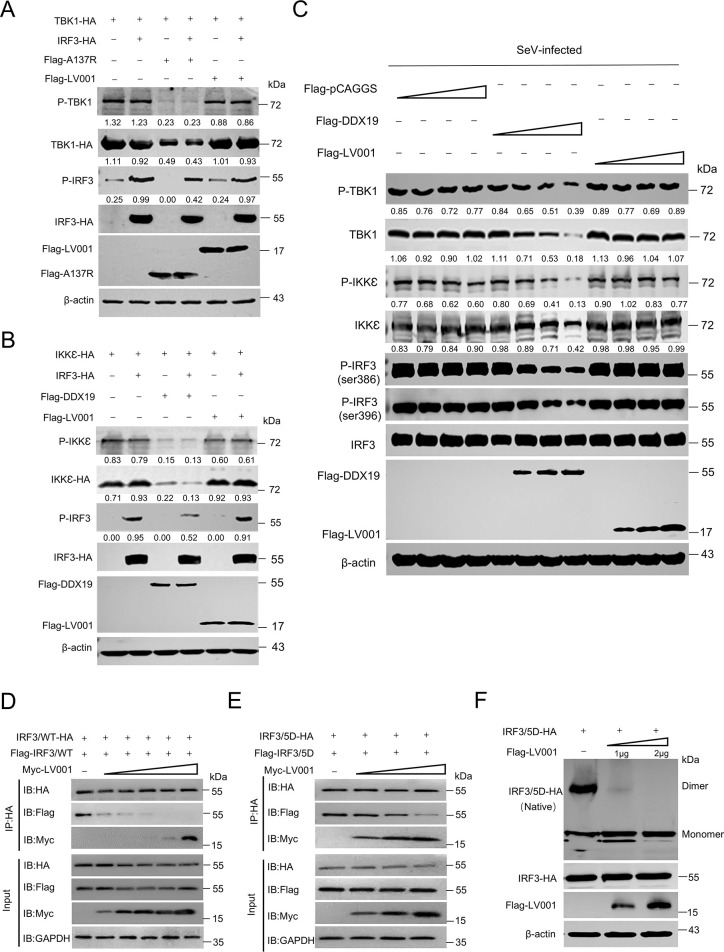
LV001 inhibits IRF3 dimerization. (A) HEK293T cells were cotransfected with FLAG-LV001, FLAG-A137R, HA-tagged IRF3, and HA-tagged TBK1 to activate IRF3. Protein extracts were obtained at 24 h post-transfection (hpt) and analyzed by immunoblotting for TBK1, phosphorylated TBK1 (p-TBK1), IRF3, and phosphorylated IRF3 (p-IRF3), with β-actin serving as a loading control. (B) RAW 264.7 cells were cotransfected with FLAG-LV001, FLAG-DDX19, HA-tagged IRF3, and HA-tagged IKKε. Protein extracts were analyzed 24 hpt by immunoblotting for IKKε, phosphorylated IKKε (p-IKKε), IRF3, and p-IRF3, with β-actin as loading control. (C) RAW 264.7 cells were transfected with increasing amounts of plasmids expressing FLAG-LV001, FLAG-DDX19 or an empty vector for 24 h, followed by stimulation with SeV at an MOI of 1 for 8 h. Proteins, including IRF3, p-IRF3 (ser386 and ser396), TBK1, p-TBK1, IKKε, and p-IKKε, were detected by immunoblotting, with β-actin as loading control. (D) HEK293T cells were transfected with IRF3/WT-HA, FLAG-IRF3/WT, or Myc-LV001 for 24 h, followed by stimulation with Sendai virus (SeV) at a multiplicity of infection (MOI) of 1 for 8 h. (E) Similar to those in panel D, HEK293T cells were transfected with IRF3/5D-HA, FLAG-IRF3/5D, or Myc-LV001 for 36 h. Proteins were extracted, and the cell lysates were immunoprecipitated with an anti-HA antibody. Cell lysates and immunoprecipitates were analyzed by immunoblotting. (F) Following a procedure similar to that in panel E, non-denaturing (native) PAGE was performed to assess IRF3 dimer formation. Cell lysates and immunoprecipitates were analyzed by immunoblotting.

IRF3 is typically activated through phosphorylation by TBK1 or IKKε [43,44]. We explored whether the interaction between LV001 and IRF3 affects IRF3 activation. Given that LV001 inhibits IRF3/5D-induced IFN-β promoter activation, it is likely that LV001 does not interfere with TBK1/IKKε-mediated IRF3 phosphorylation. To test this hypothesis, we co-transfected HEK293T or RAW264.7 cells with pFLAG-LV001 and either pMyc-TBK1, pMyc-IKKε, or pHA-IRF3. As controls, we included A137R and DDX19, known promoters of TBK1/IKKε degradation [45,46]. We then assessed TBK1, IKKε, and IRF3 protein levels and phosphorylation status by Western blotting. LV001 did not affect the protein or phosphorylated levels of these molecules (Fig 7A and 7B). Furthermore, we confirmed that endogenous TBK1, IKKε, and IRF3 phosphorylation remained unaffected by LV001 in SeV-infected or poly(I:C)-stimulated HEK293T cells (Figs 7C and S6). Phosphorylated IRF3 forms a dimer, which is essential for its nuclear translocation [47]. Since LV001 did not alter IRF3 phosphorylation, we next examined whether it affected IRF3 dimerization. Co-IP assays demonstrated that LV001 markedly impaired the interaction between IRF3 monomers, resulting in inhibition of IRF3 dimerization in a dose-dependent manner (Fig 7D and 7E). And native PAGE analysis further supported this conclusion (Fig 7F). Additionally, we also tested the interaction between IRF3 and the CBP/p300 coactivators in the presence of LV001. Our results indicated that LV001 did not affect the binding of p300 to IRF3 (S7 Fig). These findings confirm that while LV001 does not interfere with TBK1- or IKKε-mediated IRF3 phosphorylation or p300 binding to IRF3, it blocks IRF3 dimerization, thereby inhibiting its downstream signaling activity.

### LV001 blocks the nuclear translocation of IRF3

**Fig 8:**
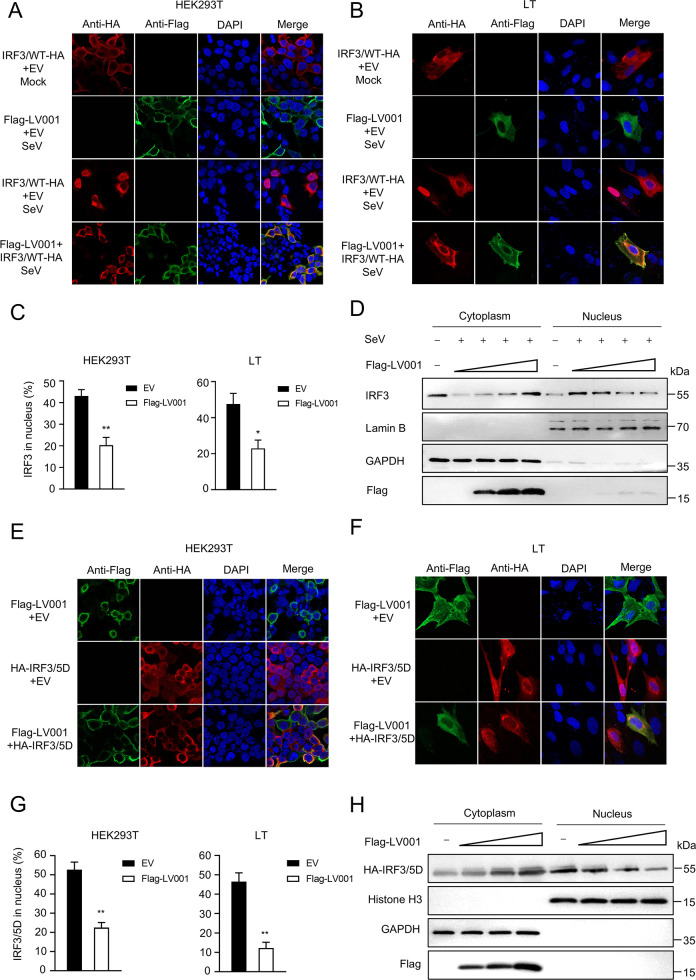
LV001 blocks the nuclear translocation of IRF3. (A, B, E, F) HEK293T or LT cells were transfected with FLAG-LV001 or an empty vector along with IRF3/WT-HA or IRF3/5D-HA for 24 h post-transfection (hpt) and then stimulated with Sendai virus (SeV) at an MOI of 1 for 8 h. The subcellular localization of IRF3 (red), FLAG-LV001 (green), and cell nuclei (blue) is observed using laser confocal microscopy. (C and G) The proportion of cells that showed IRF3 nuclear translocation was quantified from 100 cells per condition across different fields, as shown in panels A and E. Error bars represented standard errors of the means (SEMs). (D and H) HEK293T cells were transfected with plasmids encoding LV001 and IRF3/WT, infected with SeV, or transfected with plasmids encoding LV001 and IRF3/5D. IRF3 in the nuclear and cytoplasmic compartments was detected by Western blotting, with Lamin B and GAPDH serving as nuclear and cytosolic markers, respectively. Data were analyzed using Student’s *t*-test: *, *P* < 0.05; **, *P* < 0.01. ns, not significant.

After dimerization, IRF3 translocates to the nucleus to initiate IFN-β gene transcription [48,49]. Given that LV001 disrupts IRF3 dimerization, we hypothesized that it may also inhibit its nuclear translocation. To test this hypothesis, we employed laser confocal microscopy to analyze the subcellular localization of IRF3 in HEK293T and LT cells transfected with pIRF3/WT-HA, pFLAG-LV001, or an empty vector (EV). In the unstimulated group, IRF3 predominantly localized in the cytoplasm. Upon SeV stimulation, approximately 43.0% and 47.6% of IRF3 translocated to the nucleus in EV-transfected HEK293T and LT cells, respectively. However, ectopic expression of pLV001 significantly reduced nuclear localization, with only 20.0% or 22.9% of IRF3 detected in the nucleus (Fig 8A–8C). Consistently, the LV001(120–124A) mutant exhibited no effect on IRF3 nuclear translocation (S8 Fig). To confirm this effect, cytoplasmic and nuclear fractions were isolated from HEK293T cells transfected with pFLAG-LV001 or EV after SeV infection. Analysis via Western blotting revealed that nuclear-translocated IRF3 levels decreased significantly as LV001 expression increased (Fig 8D). Similarly, in the case of the constitutively active IRF3/5D, 52.0% and 46.5% of the protein translocated to the nucleus in EV-transfected HEK293T or LT cells, respectively. In contrast, only 22.0% or 12.2% of IRF3/5D was detected in the nucleus in the presence of pLV001 (Fig 8E–8G). Furthermore, as LV001 expression increased, a larger proportion of IRF3/5D was retained in the cytoplasm, resulting in reduced nuclear translocation (Fig 8H). These findings demonstrate that LV001 disrupts IRF3 dimerization, effectively blocking its nuclear translocation and thereby inhibiting downstream signaling required for IFN-β gene transcription.

### LV001 does not influence the classical nuclear localization signal (NLS) nuclear transport pathway for IRF3

**Fig 9:**
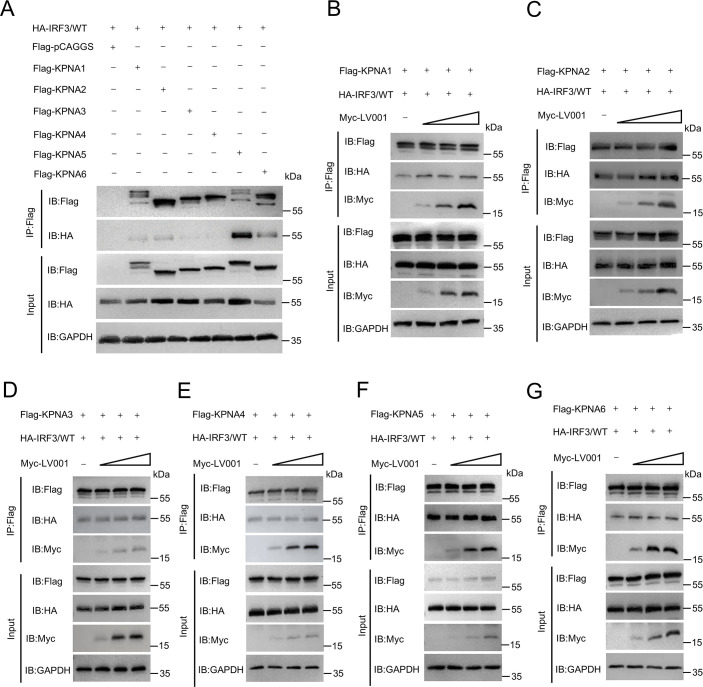
LV001 does not affect the classical nuclear localization signal (NLS) nuclear transport pathway for IRF3. (A-G) LV001 did not inhibit the interaction of IRF3 with KPNA1–6. (A) HEK293T cells were cotransfected with HA-LV001 and FLAG-tagged KPNA1-6. At 24 h post-transfection (hpt), the cell lysates were immunoprecipitated with an anti-FLAG antibody. Both whole-cell lysates and immunoprecipitates were analyzed by Western blotting to assess the interaction between IRF3 and KPNA1-6. (B-G) For further investigation, HEK293T cells were cotransfected with Myc-LV001, HA-IRF3, and FLAG-tagged KPNA1-6. At 24 hpt, the cell lysates were immunoprecipitated with an anti-FLAG antibody, and the whole-cell lysates and immunoprecipitates were analyzed using Western blotting to evaluate the effects of LV001 on the interaction of IRF3 with KPNA1-6.

Nuclear translocation of transcription factors, such as IRF3, relies on importin/karyopherin (KPNA) proteins, which recognize and bind to classical nuclear localization signals (NLS) in cargo proteins [50,51]. The human karyopherin α family comprises six primary members (KPNA1–6) that play critical roles in the nuclear translocation of IRF3. To investigate whether LV001 affects the interaction between IRF3 and KPNAs, we confirmed that IRF3 interacts with KPNA1–6. Co-immunoprecipitation assays revealed that LV001 did not disrupt this interaction (Fig 9A–9G). Additionally, we examined whether LV001 interferes with nuclear transport through the classical NLS-GFP tandem protein. Both Western blotting and confocal microscopy confirmed that LV001 did not affect the nuclear entry of cargo proteins via the KPNA-mediated pathway (S9 Fig). Collectively, these results demonstrate that LV001 inhibits IRF3 nuclear translocation by disrupting its dimerization, rather than interfering with karyopherin-mediated nuclear transport.

### The LV001 gene promotes LSDV pathogenesis *in vivo*

**Fig 10:**
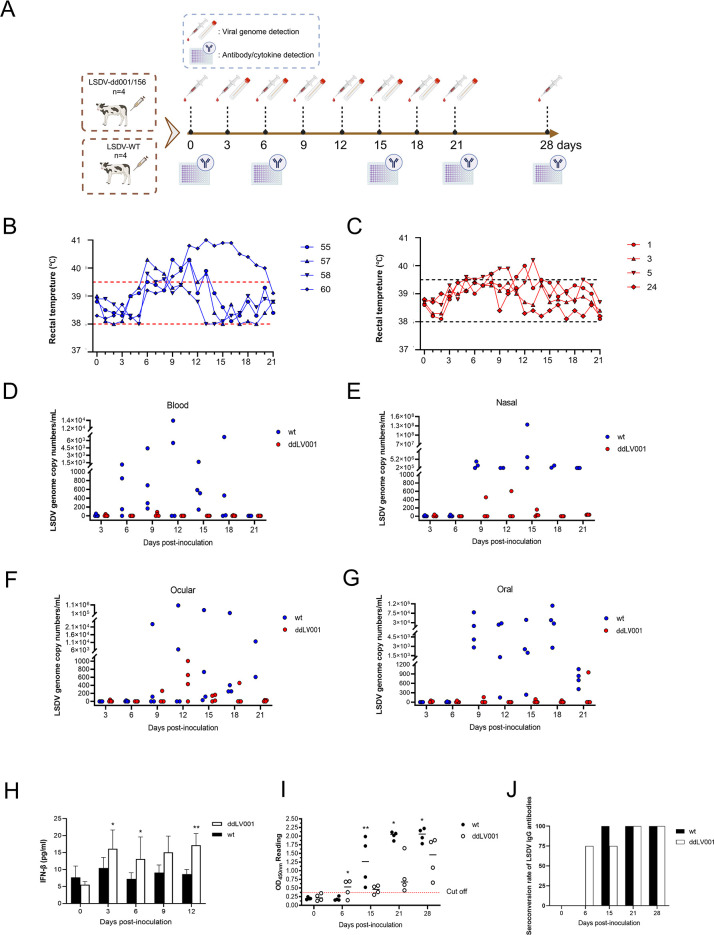
The LV001/156 gene is involved in LSDV pathogenicity in cattle. (A) Schematic diagram of the cattle infection experiment. The cattle were inoculated intradermally injection (i.n.) with LSDV-dd001/156 or LSDV-WT (n = 4) at a dose of 1 × 10^7^ TCID_50_. Schematic diagram was created with BioRender.com. The rectal temperatures of each cattle were recorded (B and C) in both groups. The viral loads in the blood (D) and different swabs (E, F and G) of each cattle in different groups were quantified by a quantitative real-time PCR. (H) The IFN-β production in the infected cattle. (I and J) The serum antibodies and rate of seroconversion of LSDV in the infected cattle. All the data were analyzed using Student’s *t*-test. *, *P* < 0.05; **P < 0.01.

Our results showed that LV001 is required for replication by negatively regulating IFN signaling *in vitro*, indicating that LV001/156 may contribute to the pathogenicity of LSDV *in vivo*. To evaluate the role of LV001 in virulence, we conducted a challenge experiment in cattle (Fig 10A). Four cattle in each group were intradermally inoculated with LSDV-dd001/156 or LSDV-WT at a dose of 10⁷ TCID₅₀. All four cattle inoculated with LSDV-WT developed fever with a maximum temperature up to 40.9°C starting from day 6 post-inoculation (Fig 10B and 10C). These animals also showed typical clinical signs of LSDV infection, including secondary nodules, depression, anorexia, and nasal discharge. In contrast, only two of the four cattle inoculated with LSDV-dd001/156 exhibited a mild increase in body temperature, which was not associated with viremia. None of these animals developed swelling at the injection site or secondary nodules during the 21-day observation period. Additionally, viral loads in the blood, ocular swabs, oral swabs, and nasal swabs of LSDV-dd001/156-infected cattle were significantly lower than those of the LSDV-WT-infected group (Figs 10D–10G and S10). To further assess the in vivo lesion-forming capacity of the two viruses, we examined the injection sites of infected cattle. No significant lesions were observed at the injection sites of LSDV-dd001/156-infected cattle, except for a minor nodule at high doses (10^6^ or 10^7^ TCID₅₀). In contrast, cattle infected with LSDV-WT showed noticeable swelling at the injection sites across all doses (S11 Fig). Collectively, these findings demonstrate that LSDV-dd001/156 exhibits significantly reduced virulence compared to LSDV-WT in cattle, highlighting the role of LV001 in the pathogenicity of LSDV *in vivo*.

### Immune responses induced by LSDV-dd001/156 infection are related to viral virulence

To confirm the link between attenuated virulence and the immune response induced by LSDV-dd001/156 infection, serum levels of IFN-β were measured. The results showed that the LSDV-dd001/156 group exhibited significantly higher IFN-β levels than the LSDV-WT group at 3, 9, and 12-day post-infection (dpi) (Fig 10H). Additionally, total LSDV-specific IgG antibodies were evaluated using an indirect enzyme-linked immunosorbent assay (ELISA). The findings revealed that the LSDV-dd001/156 group-initiated antibody production earlier (at 6 dpi) compared to the LSDV-WT group, although the latter ultimately reached higher antibody levels (Fig 10I). By the end of the experiment, 100% seroconversion of LSDV-specific IgG antibodies was observed in all experimental cattle (Fig 10J). In summary, these findings suggest that the pathogenicity of LSDV-dd001/156 in cattle is observably attenuated compared to LSDV-WT, as evidenced by elevated levels of IFN-β production and an earlier onset of antibody responses.

## Discussion

The *Poxviridae* family comprises large DNA viruses that can infect both humans and animals [52,53]. These viruses replicate in the cytoplasm and encode over 100 proteins, many of which remain poorly characterized, particularly those encoded by ITRs at the genome’s extremities. ITRs consist of identical sequences in opposing orientations, with lengths varying significantly between poxvirus species, from less than 1 kb to over 17 kb. The duplicated nature of ITR-encoded genes often results in their elevated expression due to gene dosage effects, especially for immunomodulatory proteins [11]. These proteins are integral to poxvirus immune evasion, allowing the virus to modulate or disrupt key viral-host interactions to evade immune detection and clearance. Such strategies not only enhance immune escape but also likely contribute to the broad host range observed in many poxviruses. Further exploration of ITR-encoded genes is essential to understanding their roles in immune modulation and host adaptation [7].

In this study, we show that LV001 plays a significant role in viral replication and is a late viral protein with a molecular weight of approximately 18 kDa. It seems that LV001 is not essential for the production of mature virions, although it is present in viral particles. Viral infections trigger innate immune responses, leading to the production of IFN-Is and other cytokines that combat viral invasion. IFN-Is are crucial for the inhibition of viral replication, elimination of infected cells, and initiation of adaptive immune responses. Poxviruses activate multiple host DNA-sensing pathways that converge on IRF3 to induce the expression of IFN-I and ISG, thus limiting viral replication and triggering adaptive immunity [54–56]. Because deletion of LV001 significantly reduced LSDV infectivity *in vitro*, we hypothesized that it could be an antagonist of IFN signaling after viral infection. Our findings showed that LV001 inhibits cGAS-STING and poly(dA·dT)-induced IFN-I production, supporting this hypothesis.

Although it is generally accepted that early-expressed viral proteins are key regulators of IFN production, our study revealed that LV001 is a late-expressed gene. In particular, it is a structural protein in viral particles, which is further supported by a previous study [37]. After the virus enters the host cells, the LV001 protein is likely to be released into the cytoplasm, where it quickly antagonizes the host’s innate immune response by targeting IRF3. IRF3, a key transcription factor that regulates IFN-I production in response to pathogens, is typically activated by the phosphorylation of residues 386 and 396. This phosphorylation enhances IRF3 transcriptional activity and plays a role in the immune response. In resting cells, IRF3 resides in the cytoplasm, but becomes phosphorylated by TBK1 and IKKε upon viral infection [57]. Following phosphorylation, IRF3 homodimerizes, translocates to the nucleus, binds to target genes, and activates transcription by interacting with CBP/p300 coactivators. Our results indicated that LV001 does not affect IRF3 phosphorylation or its interaction with p300. Instead, LV001 disrupted IRF3 dimerization without affecting the phosphorylation status. Phosphorylation triggers a conformational change that exposes the DBD and IAD, allowing IRF3 to form homodimers in the IAD region [47,58–60]. We showed that LV001 directly interacts with the IAD domain of IRF3. Therefore, LV001 may preferentially bind to phosphorylated IRF3 because the IAD region is exposed to IRF3 phosphorylation.

The classical nuclear import pathway is the best understood system for transporting macromolecules between the cytoplasm and nucleus [51]. In this pathway, proteins containing a classical basic NLS are imported by a heterodimeric import receptor composed of karyopherin importin β, which mediates interactions with the nuclear pore complex, and importin α, which binds directly to the NLS. Classical NLS typically consist of a short sequence of basic amino acids, continuous or separated by a 10–12 amino acid linker region. These signals are recognized by one or more of the seven importin α subunits, with KPNA1–6 being the main subunit [61]. Many transcription factors, such as NF-κB, STAT1, and members of the IRF family, contain NLSs that are transported to the nucleus through this pathway [62–64]. After recognizing its cargo, importin α binds to importin β1 (KPNB1) to form the cargo-importin α-importin β1 complex, which is then transported to the nucleus via the nuclear pore complex [65]. Within the nucleus, the dissociation of the complex is facilitated by the small G protein Ran in its GTP-bound form, which releases cargo molecules [66]. In this study, we showed that IRF3 interacts with KPNA1–6 at varying strengths, whereas LV001 did not affect these interactions. Previous studies have established that poxvirus immune modulators interact with host antiviral factors in a species-specific manner [67–69]. A prime example is the VACV E3 and K3 proteins, which exhibit species-dependent inhibition of Protein Kinase R (PKR), contributing to poxvirus host range determination. Phylogenetic evidence shows the PKR kinase domain has undergone accelerated evolution in rodents, leading to structural divergence across species [69]. This evolutionary arms race necessitates poxvirus adaptation to effectively overcome host defenses. While KPNAs are generally well-conserved across species, it remains unknown whether LV001 similarly fails to inhibit bovine KPNA-mediated IRF3 nuclear translocation as observed with human KPNAs.

Bioinformatics analysis suggests that LV001 encodes a Bcl-2-like fold, showing high homology to the VACV B14 protein. It belongs to the Bcl-2-like protein family, which includes well-characterized immune modulators such as N1, A46, C6, A52, and K7. These proteins are known to antagonize key signaling pathways, including those leading to IFN and NF-κB activation [28–31,70–72]. However, LV001 exhibits unique features distinguishing it from its counterparts. Unlike the early-expressed proteins in this family, LV001 is a late-expressed protein encoded by two copies located within the terminal regions of the genome. This genomic positioning and late expression suggest a specialized function distinct from the early-stage immunomodulatory roles of other Bcl-2-like proteins. Despite shared structural homology, the mechanisms by which these proteins inhibit innate immunity vary significantly. For instance, A52 and B14, which also possess Bcl-2-like folds, specifically target the NF-κB signaling pathway without affecting apoptosis [32]. N1, another NF-κB antagonist, uniquely exhibits dual functionality by also inhibiting apoptosis, though the precise mechanism remains unclear [73]. C6 primarily targets the IFN-I signaling pathway, with low structural homology to LSDV001 [30,31]. This functional diversity within the Bcl-2-like protein family underscores their adaptability in modulating host immune responses. LV001 appears to leverage the Bcl-2 scaffold for a distinct role, potentially aligned with late-stage viral processes, setting it apart from other family members. These observations highlight the evolutionary plasticity of the Bcl-2 fold, enabling the fine-tuning of immune evasion strategies across different viruses. LV001 represents a specialized adaptation within this framework, warranting further exploration to uncover its precise mechanism and contribution to LSDV pathogenesis.

As a negative regulator of IFN-I and ISGs production (Fig 10), the role of LV001 in LSDV virulence was also confirmed *in vivo*. The cattle inoculated with LSDV-dd001/156 did not show obvious clinical symptoms, maintained a relatively normal body temperature, and showed reduced viral shedding (Fig 9). In contrast, cattle infected with the LSDV/China/Xinjiang/2019 strain showed classic LSD symptoms, suggesting that the virulence of the LV001 deletion strain was significantly lower than that of the parental strain. Cattle infected with the LV001deletion mutant produced higher levels of IFN-β, indicating that LV001, as a structural protein and IFN-I negative regulator, primarily modulates cellular immunity, which subsequently shapes the humoral immune response.

**Fig 11:**
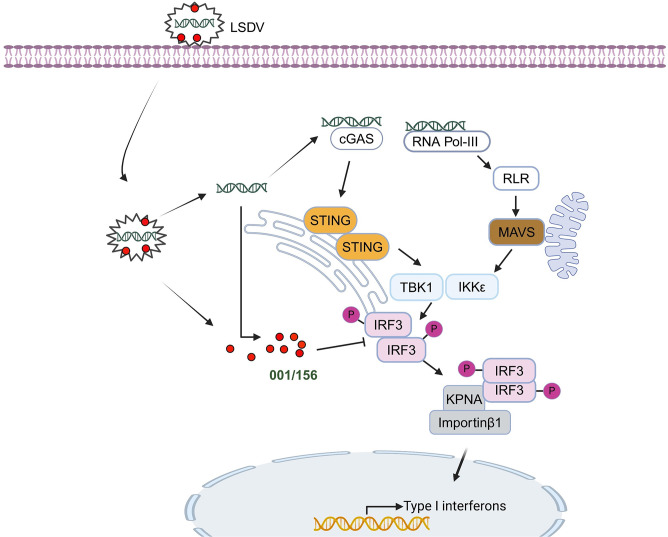
Schematic model of the LV001/156 protein that negatively regulates IFN-β production during LSDV infection. LSDV dsDNA can be sensed by cGAS, a cytosolic DNA sensor, or transcribed into dsRNA by RNA polymerase III, leading to the activation of the type I interferon (IFN-I) signaling pathway. However, to evade the innate immune response, the viral protein LV001/156 interact with IRF3, impairing its dimerization. This interaction reduces or blocks importin family-mediated nuclear entry, which is dependent on IRF3, thus inhibiting its function as a transcription factor and decreasing INF-I production during LSDV infection. Figure was created with BioRender.com.

In conclusion, to the best of our knowledge, this is the first study to confirm that LV001 significantly influences LSDV replication. It is a late-expressed protein that interacts with IRF3, disrupting dimerization, and inhibiting IFN-β production (Fig 11). These findings increase our understanding of how genes located in the ITRs of poxviruses contribute to immune evasion and shed light on the mechanisms by which LV001 regulates LSDV virulence.

## Materials and methods

### Ethics statement

Experiments involving LSDV infection in animals were performed under animal biosecurity level 3 (ABSL-3) conditions at Spirit Jinyu Biological Pharmaceutical Co. Ltd. Animal experiments were conducted in compliance with the Animal Welfare Act and the Guide for the Care and Use of Laboratory Animals, and were approved by the Laboratory Animal Welfare Committee of the Harbin Veterinary Research Institute (HVRI) of the Chinese Academy of Agricultural Sciences (approval number: 241108–03-GR).

### Cell culture and materials

Primary lamb testicle (LT) cells, HEK293T and RAW264.7 cells were cultured in modified Eagle’s minimal essential medium (HyClone, Thermo Scientific, MA, USA) supplemented with 10% fetal bovine serum (FBS; Sigma‒Aldrich, St. Louis, MO, USA), penicillin (100 U/mL), and streptomycin (100 μg/mL) (HyClone, USA) at 37°C in a humidified atmosphere containing 5% CO₂. HEK-293T, HeLa, and MDBK cells were maintained in Dulbecco’s modified Eagle’s medium (Life Technologies, Grand Island, NY, USA) supplemented with 10% or 5% FBS.

The LSDV/China/Xinjiang/2019 strain, referred to as LSDV or LSDV-WT in this study, was provided by the HVRI of the Chinese Academy of Agricultural Sciences. Virus titration was expressed as the dose infecting 50% of tissue culture parallels per milliliter (TCID₅₀/mL) or as its decimal logarithm.

### Antibodies and reagents

The following mouse monoclonal antibodies were used: anti-FLAG (A00187, GenScript), anti-GST (66001–2-IG, Proteintech), and anti-GFP (PTM-5180, PTM BIO). The rabbit monoclonal and polyclonal antibodies included: anti-FLAG (PTM-5577, PTM BIO), anti-GAPDH (PTM-5375, PTM BIO), anti-histone H3 (PTM-1001RM, PTM BIO), and anti-His (PTM-5614, PTM BIO), all purchased from Hangzhou PTM BIO. Additional antibodies used were: anti-Myc (A00172, GenScript), anti-TBK1 (38066S, Cell Signaling Technology), anti-phospho-TBK1 (5483S, Cell Signaling Technology), anti-IKKε (D20G4, Cell Signaling Technology), and anti-phospho-IKKε (Ser172) (D1B7, Cell Signaling Technology). Other antibodies included: anti-IRF3 (ab68481, Abcam), anti-phospho-IRF3 (ab76493, Abcam), anti-HA (ab9110, Abcam), and anti-lamin B (bs-23709R, Bioss). The polyclonal antibody against LV001 was generated in-house by expressing the LV001 protein in a prokaryotic system followed by mouse immunization.

Horseradish peroxidase (HRP)-conjugated goat anti-rabbit IgG (A0545) and goat anti-mouse IgG (A4416) were obtained from Sigma-Aldrich. FITC-conjugated goat anti-mouse IgG (ZF-0312) and TRITC-conjugated goat anti-mouse IgG (ZF-0317) were purchased from Beijing Zhongshan Jinqiao Biotechnology. Protein A/G-agarose (A10001M) was from Abmart. JetPRIME transfection reagent (101000046) was from Polyplus Transfection (Strasbourg, France). Poly(dA·dT) was obtained from InvivoGen. DAPI was purchased from Invitrogen (Eugene, OR, USA).

### Plasmid construction

The ORF of LV001 was amplified from the genome of the parent LSDV/China/Xinjiang/2019 strain (GenBank no.: OP508345.1), cloned, and inserted into the pCAGGS vector containing FLAG, Myc, or HA tags. The VACV C6 and N1L constructs (GenBank no.: AY243312.1) were created by cloning codon-optimized and synthesized sequences into the pCAGGS vector, with a FLAG tag fused to the 3’ end. Plasmids encoding cGAS (GenBank no.: NM_138441.3), STING (GenBank no.: MF622062.1), TBK1 (GenBank no.: NM_013254.4), and IRF3/WT (GenBank no.: NM_001571.6) were constructed by inserting synthesized sequences into the pCAGGS vector, with Myc or HA tags at the 3’ end. The IRF3/5D mutant was constructed as previously described [74]. The ORFs of KPNA1-KPNA6 were amplified, cloned, and inserted into the pCAGGS vector using a FLAG tag. The NLS-GFP-GST construct, containing the core sequence of the NLS from the SV40 large T antigen [75], was generated as previously described [76]. All constructed plasmids were confirmed by sequencing.

### Recombinant LSDV construction

LSDV recombinants were generated by homologous recombination using the parental LSDV genome and a recombination transfer vector, as previously described. The p11 promoter was used to drive EGFP expression and served as a screening cassette. The upstream and downstream sequences of LV001/156, measuring 0.45 kb and 0.43 kb, respectively, were designed as homologous recombination arms. All sequences were synthesized by GenScript Biotech Corporation and subsequently cloned and inserted into the pUC57 vector. The nucleotide sequences of the left homologous arm corresponded to nucleotides 709–1165 and 169641–150097 in the LSDV/China/Xinjiang/2019 strain (GenBank no.: OP508345.1), whereas those of the right homologous arm corresponded to nucleotides 275–705 and 150101–150531, respectively.

To generate a revertant LSDV r001/156-Myc strain expressing the Myc-tagged LV001 protein, a recombinant vector was constructed by replacing the p11 promoter driving the EGFP ORF with the sequence of a C-terminally Myc-tagged LV001 ORF. Various LV001/156 recombinants of LSDV were produced by homologous recombination between the parental LSDV genome and recombination transfer vectors using transfection and infection procedures, as previously described. LT cells were infected with the LSDV/China/Xinjiang/2019 strain, and 2 hours post-infection, cells were transfected with the constructed transfer vectors using the JetPRIME transfection reagent (Polyplus Transfection, Strasbourg, France). After 72 h, the recombinant viral infection was confirmed by fluorescence microscopy. Recombinant viruses were purified through 4–6 rounds of plaque purification to ensure homogeneity. Final isolates were comprehensively characterized by PCR (primers listed in S1 Table), sequencing, and Western blot analysis. Validated recombinants were then amplified and titrated on LT cells for experimental use.

### LSDV propagation and purification

LSDV was propagated in LT cells cultured in T225 flasks. Upon reaching 70–80% cytopathic effect (CPE), the culture supernatant was discarded, and for cell-associated virus extraction, adherent cells were scraped, washed twice with PBS, and pelleted by centrifugation at 1,200 × g for 10 min. The pellet was resuspended in 10 mM Tris-HCl (pH 9) and subjected to three freeze-thaw cycles. After centrifugation at 800 × g for 5 min (4°C), the supernatant was collected. This step was repeated twice, and the combined supernatants (~2 mL) were used for further purification. The clarified supernatant was underlaid with 36% sucrose (in 10 mM Tris-HCl, pH 9) and centrifuged at 32,900 × g for 80 min (4°C). The resulting pellet was resuspended in 1 mL of 1 mM Tris-HCl (pH 9) and layered onto a discontinuous sucrose gradient (28%, 32%, 36%, 40%, and 70% sucrose; 2 mL each). Following centrifugation at 26,000 × g for 50 min (4°C), the virus band at the 40%–70% sucrose interface was collected, diluted in 10 mL of 1 mM Tris-HCl (pH 9), and pelleted at 15,000 × g for 30 min. The final pellet was resuspended in 80 µL of 1 mM Tris-HCl (pH 9) and stored at −70°C.

### RNA-seq data preprocessing and analysis

Raw sequencing reads were quality-trimmed and adapter-filtered using Cutadapt, followed by additional filtering with custom Perl scripts to remove: 1) reads containing ≥5 bp of adapter sequence (with both paired reads discarded if either was contaminated), 2) reads with >15% of bases having Phred scores ≤19, and 3) reads containing >5% ambiguous bases. Reads were aligned to the Ensembl reference genome (indexed with Bowtie2 v2.2.3) using HISAT2 v2.1.0, with alignments visualized in IGV. Differential expression analysis was performed with DESeq2 (adjusted p < 0.05, |log2FC| > 1), followed by functional enrichment analysis using ClusterProfiler (adjusted p < 0.05).

### Dual-luciferase reporter assays

To assess the binding activity of various promoters, firefly luciferase reporter plasmids (IFN-β-Luc, ISRE-Luc, NF-κB-Luc, or IRF3-Luc), along with Renilla luciferase reporter plasmids (pRL-TK) and other expression plasmids, were cotransfected into HEK293T cells in 48-well plates. After 24 h of transfection, cells were lysed using passive lysis buffer, and luciferase activity was measured using the Dual-Luciferase Reporter Assay System (E1910, Promega) according to the manufacturer’s protocol. Firefly luciferase activity was normalized to that of Renilla luciferase, and the results were presented as the ratio of Fluc to Rluc. Each experiment was conducted in triplicate.

### RNA extraction and RT-qPCR

Total RNA from cells subjected to specific treatments or viral infections was extracted using an RNAprep Pure Cell/Bacteria Kit (catalog no. DP430, Tiangen) and reverse-transcribed into cDNA using HiScript II Q RT SuperMix for qPCR (catalog no. R223-01, Vazyme). The resulting cDNAs from HEK293T or MDBK cells were used as templates for RT-qPCR using SYBR Premix Ex Taq (catalog no. RR820A, TaKaRa) to quantify the mRNA levels of human or bovine IFN-β, ISG15, and ISG56. The glyceraldehyde-3-phosphate dehydrogenase (GAPDH) gene was used as an internal control. All primers used are listed in S1 Table.

### Co-IP assay

For coimmunoprecipitation, expression plasmids were transfected or cotransfected into HEK293T cells for 24 h. The cells were lysed in ice-cold NP-40 lysis buffer (P0013F; Beyotime). After centrifugation of the collected lysates, proteins were isolated by incubating the supernatants with an appropriate amount of ANTI-FLAG M2 Affinity Gel (A2220, Sigma) or EZview Red Anti-HA Affinity Gel (E6779, Sigma) overnight at 4°C. The beads were then washed five times with phosphate-buffered saline containing 0.1% Tween and boiled in 4 × sample buffer before SDS-PAGE analysis.

### GST pulldown assay

The proteins were obtained from Frdbio Bioscience and Technology Co., Ltd. (Wuhan, China). Briefly, purified GST-IRF3 or GST proteins, together with 6 × His-LV001, were immobilized on BeyoGold GST-tag Purification Resin (P2251, Beyotime), which was prepared by washing with binding buffer (pH 8.0; 50 mM Tris-HCl, 200 mM NaCl, 1 mM EDTA, 1% (v/v) NP-40, 1 mM DTT, and 10 mM MgCl₂). Proteins bound to the resin were eluted using elution buffer (pH 8.0; 50 mM Tris-HCl, 400 mM NaCl, 1 mM EDTA, 1 mM DTT, and 50 mM GSH), and the eluted proteins were treated with 1 × SDS loading buffer. Proteins were detected by Western blotting with anti-GST or anti-His polyclonal antibodies.

### Immunofluorescence assay

HEK293T cells were transfected with expression plasmids or EVs (0.5 µg each) for 24 h, followed by fixation with 4% paraformaldehyde (BL539A, Biosharp) for 30 min at room temperature. After two washes with PBS, the cells were permeabilized with 0.15% Triton X-100 for 15 min at room temperature. The cells were then incubated with a mouse anti-FLAG monoclonal antibody (1:200) for 1.5 h at 37°C. The cells were then incubated with Alexa Fluor 633-conjugated goat anti-mouse IgG (H + L) (1:100) or Alexa Fluor 488-conjugated donkey anti-rabbit IgG (H + L) (1:100) for 45 min and washed with PBS. Subsequently, the nuclei were stained with 4′,6-diamidino-2-phenylindole (DAPI) (1:1,000) (Invitrogen, Eugene, OR, USA) and visualized using a laser confocal microscope (LSM880, Zeiss).

For IRF3 nuclear translocation experiments, HEK293T or LT cells were cotransfected with LV001 and HA-IRF3 expression plasmids or EVs (0.5 µg each) for 24 h and then fixed in 4% paraformaldehyde (BL539A, Biosharp) for 30 min at room temperature. After washing twice with PBS, the cells were permeabilized with 0.15% Triton X-100 for 15 min. The cells were treated with a mouse anti-FLAG monoclonal antibody (1:200, M4439-100UL; Sigma‒Aldrich) or a rabbit anti-HA antibody (1:200) for 1.5 h at 37°C. After incubation, cells were treated with Alexa Fluor 633-conjugated goat anti-mouse IgG (H + L) antibody (1:100) or Alexa Fluor 488-conjugated donkey anti-rabbit IgG (H + L) antibody (1:100) for 45 min and washed with PBS. Finally, the nuclei were stained with DAPI (1:1,000) (Invitrogen, Eugene, OR, USA) and observed using a laser confocal microscope (LSM880, Zeiss).

### ELISA

Human and bovine IFN-β levels in cell culture supernatants or serum were determined using ELISA kits from mlBIO Biotechnology (ml027494–2/ml036570–2; Shanghai mlBIO), according to the manufacturer’s instructions. Serum LSDV-specific IgG levels were tested using ELISA kits from mlBIO Biotechnology (Beijing, CAHIC).

### AlphaFold2-based modeling of protein interactions

The structural interaction between viral protein LV001 and host protein IRF3 was predicted using AlphaFold2. Full-length sequences of both proteins were input into the model, which generated multiple sequence alignments and produced five candidate structures. The top-ranked model was selected based on the highest predicted TM-score (pTM) and interface predicted TM-score (ipTM), with an ipTM of 0.81 indicating high confidence in the protein-protein interface prediction. Structural visualization and interface analysis were conducted using PyMOL and PDBePISA, revealing key interacting residues and an estimated interface area of 1100 Å^2^.

### Preparation of nuclear and cytoplasmic extracts

Cells transfected with the plasmids were processed according to the manufacturer’s instructions using a Nuclear and Cytoplasmic Protein Extraction Kit (P0028; Beyotime, China).

### Animal experiments

The virulence and pathogenesis of LSDV dd001/156 were assessed and compared with those of the parental strain LSDV/China/Xinjiang/2019. In this study, LSDV-seronegative cattle were intradermally inoculated with LSDV-dd001/156 or LSDV/China/Xinjiang/2019. The cattle were monitored daily for 28 days, during which time rectal temperatures and clinical signs were recorded. Blood samples and nasal, oral, and ocular swabs were collected at 3, 6, 9, 12, 15, 18, and 21-day post-challenge to detect viral DNA and serum-neutralizing antibody levels. Viral loads were determined using quantitative PCR (qPCR), and LSDV serum-neutralizing antibodies were measured in LT cells as previously described [38].

One bull calf was inoculated with both LSDV-dd001/156 and the LSDV/China/Xinjiang/2019 strains at doses of 10^2^, 10^3^, 10^4^, 10^5^, or 10^6^ 50% of tissue culture infective doses (TCID_50_) in the left and right thorax and abdomen, respectively. The cattle were observed daily for 2 weeks after inoculation.

### Statistical analysis

All *in vitro* experiments were performed at least thrice. Data are presented as mean ± standard deviation (SD). Statistical significance between groups was assessed using t-test/ANOVA performed using GraphPad Prism (version 9.0, San Diego, CA, USA). * *P* < 0.05, ** *P* < 0.01, *** **P* *< 0.001, and **** **P* *< 0.0001 were considered statistically significant; “ns” indicates no significant difference.

## Supporting information

S1 Table Primers and oligonucleotides used in this study. This table lists all primers and oligonucleotide sequences used for PCR amplification, cloning, and other molecular biology applications in this study.
(DOCX)

S1 Fig Nucleotide sequences of the wild-type LV001 and revertant LV001 (rLV001) at the 5′ region. (A) Nucleotide sequence of the LV001 gene and its flanking regions in wild-type LV001. The LV001 coding region is highlighted in light blue. The start codon of LV001 is shown in red, and a portion of the LV002 sequence is indicated in blue. (B) Nucleotide sequence of the LV001 gene and its flanking regions in revertant LV001 (rLV001). The LV001 coding region is highlighted in light blue, and the MYC tag is indicated in uppercase letters. The start codon of LV001 is shown in red, and a portion of the LV002 sequence is indicated in blue. Italicized sequences represent non-coding regions.
(TIF)

S2 Fig Screening and identification of the LSDV-dd001/156 mutant. (A) Final screening of LSDV-dd001/156 mutant. The LSDV-dd001/156 mutant was obtained through multiple rounds of EGFP fluorescence plaque screening. (B) Identification of recombinant viruses by Western blotting. LT cells were infected with LSDV-WT, -rLV001, -sdLV001/156, and -ddLV001/156 at an MOI of 0.5. After 48 h, LT cells showing 70 − 80% CPE were scraped and centrifuged at 800 × *g*, and cell pellets were lysed with RIPA buffer. The lysates were mixed with 4 × SDS sample buffer, heated to 95°C for 15 min, and analyzed by Western blotting. (C) The rLV001 recombinant virus was identified by PCR and sequencing. Identification primers were used to apply LV001 and its flanking nucleic acid and LV001/156-Myc gene.
(TIF)

S3 Fig Multiple sequence alignment of LV001 and its orthologs across poxvirus species. Orthologs from genera such as *Orthopoxvirus* (VACV, GenBank: AY243312; MPXV, GenBank: OZ256747) and *Capripoxvirus* (GTPV, GenBank: MH381810; SPPV, GenBank: PP886239) were included. Representative members from both genera were selected for comparison. Conserved Bcl-2-like protein motifs are indicated by red boxes.
(TIF)

S4 Fig LV001 inhibits LSDV-induced IFN-β production. MDBK cells infected with LSDV-dd001/156, LSDV-sd001/156, LSDV r001-156-Myc revertant, or LSDV-WT (MOI = 5) were treated with (D–F) or left untreated (A–C) AraC. IFN-β, ISG15, and ISG56 mRNA levels were quantified by RT-qPCR at 24 hpi.
(TIF)

S5 Fig LV001-IRF3 interaction analysis. (A) Coomassie-stained SDS-PAGE of purified His-LV001 and GST-IRF3 expressed in *E. coli.* (B) SPR sensorgrams showing dose-dependent LV001 binding to immobilized IRF3 with calculated K_D_ values.
(TIF)

S6 Fig LV001 does not affect IRF3 phosphorylation. (A) Immunoblot analysis of HEK293T cells transfected with FLAG-LV001 or empty vector, probing for IRF3, p-IRF3 (Ser386/396), TBK1, p-TBK1, IKKε, and p-IKKε. (B) Same analysis as in (A) following poly(I:C) stimulation (12 h). β-actin served as a loading control.
(TIF)

S7 Fig LV001 does not disrupt IRF3–p300/IBID interaction. (A) Co-IP of HA-IRF3 and FLAG-p300/IBID in HEK293T cells expressing Myc-LV001 or EGFP control. (B) Dose-response co-IP with increasing Myc-LV001 (0.5-4 μg). Input lysates and IPs were analyzed by Western blot.
(TIF)

S8 Fig LV001(120-124A) exhibits impaired inhibition of IRF3/5D nuclear translocation inhibition. (A and C) Confocal microscopy of HEK293T/LT cells co-expressing IRF3/5D-HA with FLAG-LV001 or FLAG-LV001(120–124A). Nuclei (DAPI, blue), IRF3 (red), and LV001 (green) are shown. (B and D) Quantification of IRF3 nuclear translocation from ≥100 cells/condition. Data are represented mean ± SEM (*p < 0.05, **p < 0.01, ***p < 0.001 by Student’s t-test).
(TIF)

S9 Fig LV001 does not block the nuclear entry of NLS-guided cargo proteins. (A) HEK293T cells were transfected with FLAG-LV001 or an empty vector along with NLS-GFP-GST for 24 hpt. The subcellular localization of IRF3 (green), FLAG-LV001 (red), and cell nuclei (blue) was visualized using laser confocal microscopy. Nuclear translocation of NLS-GFP-GST was quantified from 100 cells per condition in different fields. Error bars denoted standard errors of the mean. (B) HEK293T cells were cotransfected with increasing amounts of FLAG-LV001 or an empty vector along with NLS-GFP-GST (1.5 μg). The distribution of NLS-GFP-GST in the nuclear and cytoplasmic compartments was detected by Western blotting. Histone H3 and GAPDH were used as markers for the nuclear and cytosolic compartments, respectively.
(TIF)

S10 Fig Viral shedding in LSDV-infected cattle. (A–D) Cattle infected with LSDV-dd001/156 exhibited detectable viral shedding at six different time points. (E–H) Cattle in the LSDV-WT group exhibited viral shedding at six corresponding time points.
(TIF)

S11 Fig Comparison of the lesion-forming capacity of the two viruses *in vivo.* (A) Different doses of the two viruses were administered intradermally on opposite sides of the body to continuously monitor lesion formation. (B) Comparison of lesion counts following inoculation with the two viruses over the monitoring period. Photographs were taken by the authors during the animal experiments.
(TIF)
