# Supplementary material for: Lumpy skin disease virus 001/156 protein is a virulence factor that suppresses interferon production through impairing IRF3 dimerization
Source: PLoS Pathog. 2025 Jul 23;21(7):e1013362. doi: 10.1371/journal.ppat.1013362 (PMC12313062; doi:10.1371/journal.ppat.1013362)
Supplement: S1 Table — This table lists all primers and oligonucleotide sequences used for PCR amplification, cloning, and other molecular biology applications in this study. (DOCX) [file ppat.1013362.s001.docx]

| **Primers** | | **Sequences** |
| --- | --- | --- |
| Bovine IFN-β F | 5'-ccaacaacgtcagagcctt-3' | |
| Bovine IFN-β R | 5’-gctgttcttgcttcatctcct-3' | |
| Bovine β-actin F | 5'-TCCAGCCTTCCTTCCTGGGCAT-3' | |
| Bovine β-actin R | 5'-GGACAGCACCGTGTTGGCGTAGA-3' | |
| Bovine ISG15 F | 5'-CCATGACGGTATCCGAGCTG-3' | |
| Bovine ISG15 R | 5'-GGGCCTCCCTTCAAAAGACA-3' | |
| Bovine ISG56-F | 5'-TGGACTGTGAGGAAGGATGG-3' | |
| Bovine ISG56-R | 5'-AGGCGATAGACAACGATTGC-3' | |
| Human GAPDH-F | 5'-TCATGACCACAGTCCATGCC-3' | |
| Human GAPDH-R | 5'-GGATGACCTTGCCCACAGCC-3' | |
| Human IFN-β-F | 5'-CAAATTGCTCTCCTGTTGTGCTTC-3' | |
| Human IFN-β-R | 5'-AATGCGGCGTCCTCCTTCT-3' | |
| Human ISG15-F | 5'-CTGCAACCATGAGTGAGAA-3' | |
| Human ISG15-R | 5'-CCTTTGAGGTGCTTTAGATAG-3' | |
| Human ISG54-F | 5'-CTGCAACCATGAGTGAGAA-3' | |
| Human ISG54-R | 5'-CCTTTGAGGTGCTTTAGATAG-3' | |
| Human ISG56-F | 5'-TACAGCAACCATGAGTACAA-3' | |
| Human ISG56-R | 5'-TCAGGTGTTTCACATAGGC-3' | |
| LV001 jd F | 5'-ACGAGGTCTCGAAGCAATACC-3' | |
| LV001 jd R | 5'-TCTTCCGGCAACTATGTCTACCG-3' | |
| LV156 jd F | 5'-AGGATTAGTTGAAAGGATGATG-3' | |
| LV156 jd R | 5'-GCCAATTAAACCTGTAAATGGATACT-3' | |

**S1 TABLE Primers and oligonucleotides used in this study.**
