# Supplementary figures and images for: Lumpy skin disease virus 001/156 protein is a virulence factor that suppresses interferon production through impairing IRF3 dimerization
Source: PLoS Pathog. 2025 Jul 23;21(7):e1013362. doi: 10.1371/journal.ppat.1013362 (PMC12313062; doi:10.1371/journal.ppat.1013362)

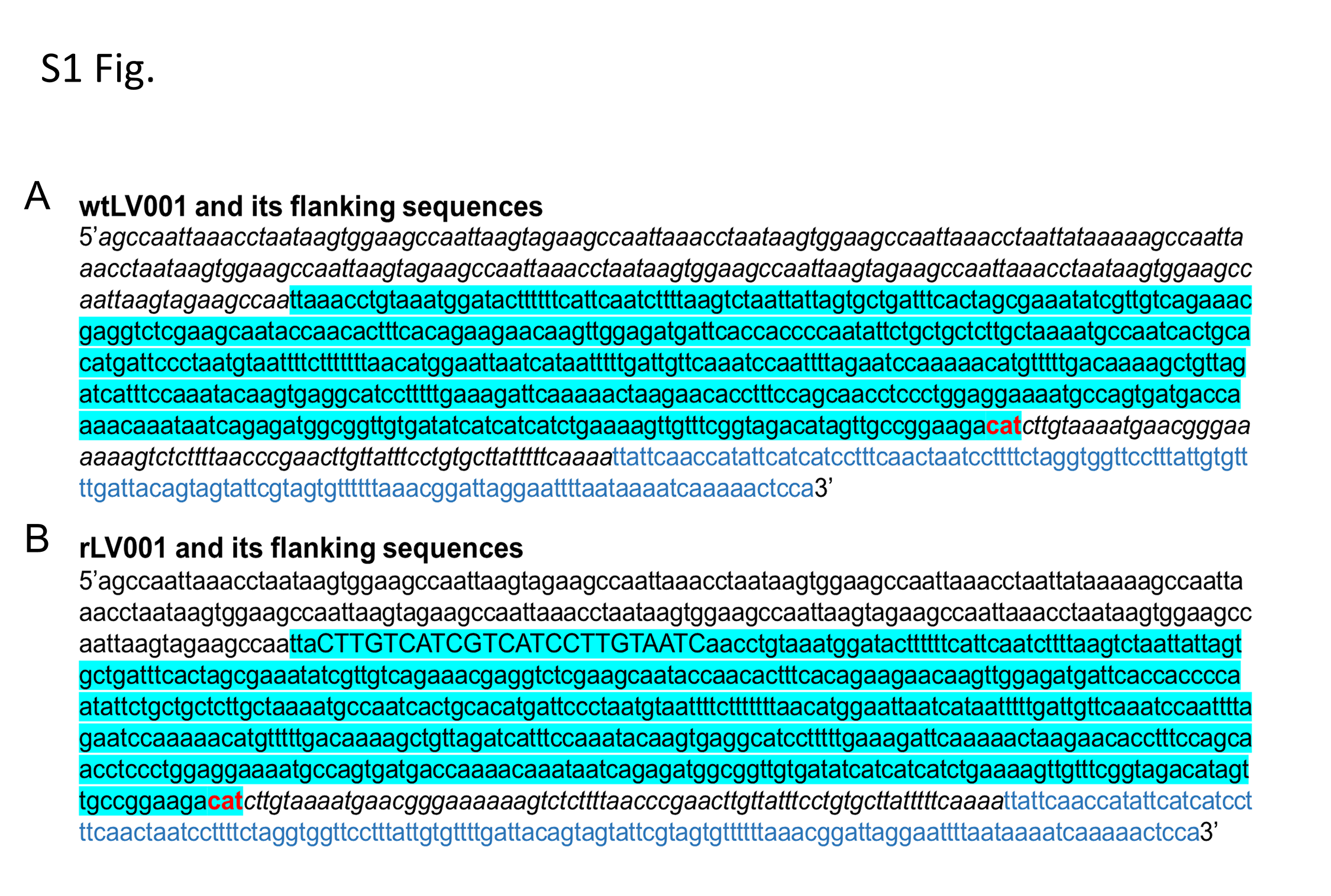

Supplement: S1 Fig — (A) Nucleotide sequence of the LV001 gene and its flanking regions in wild-type LV001. The LV001 coding region is highlighted in light blue. The start codon of LV001 is shown in red, and a portion of the LV002 sequence is indicated in blue. (B) Nucleotide sequence of the LV001 gene and its flanking regions in revertant LV001 (rLV001). The LV001 coding region is highlighted in light blue, and the MYC tag is indicated in uppercase letters. The start codon of LV001 is shown in red, and a portion of the LV002 sequence is indicated in blue. Italicized sequences represent non-coding regions. (TIF) [file ppat.1013362.s002.tif]

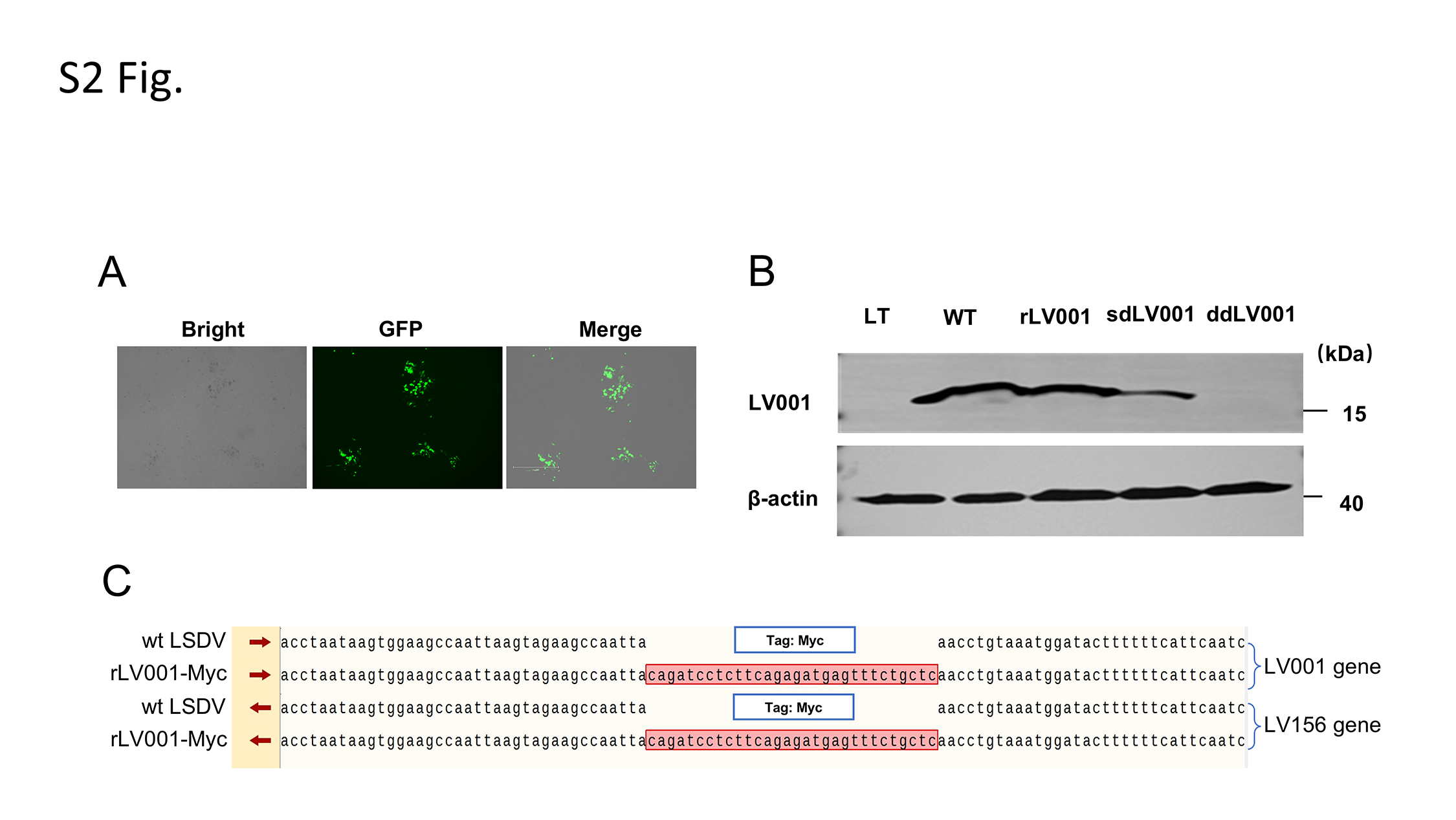

Supplement: S2 Fig — (A) Final screening of LSDV-dd001/156 mutant. The LSDV-dd001/156 mutant was obtained through multiple rounds of EGFP fluorescence plaque screening. (B) Identification of recombinant viruses by Western blotting. LT cells were infected with LSDV-WT, -rLV001, -sdLV001/156, and -ddLV001/156 at an MOI of 0.5. After 48 h, LT cells showing 70 − 80% CPE were scraped and centrifuged at 800 × g, and cell pellets were lysed with RIPA buffer. The lysates were mixed with 4 × SDS sample buffer, heated to 95°C for 15 min, and analyzed by Western blotting. (C) The rLV001 recombinant virus was identified by PCR and sequencing. Identification primers were used to apply LV001 and its flanking nucleic acid and LV001/156-Myc gene. (TIF) [file ppat.1013362.s003.tif]

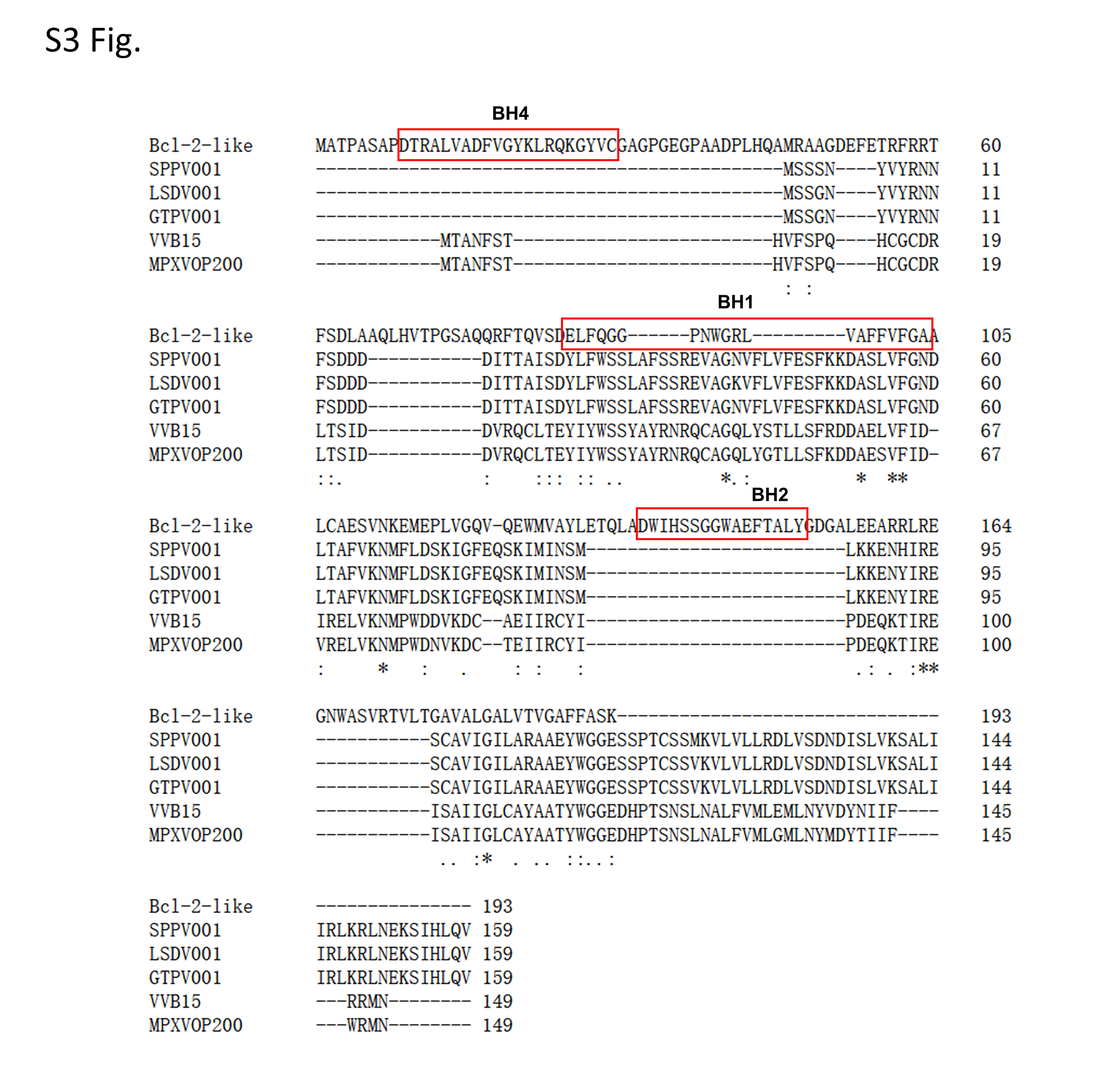

Supplement: S3 Fig — Orthologs from genera such as Orthopoxvirus (VACV, GenBank: AY243312; MPXV, GenBank: OZ256747) and Capripoxvirus (GTPV, GenBank: MH381810; SPPV, GenBank: PP886239) were included. Representative members from both genera were selected for comparison. Conserved Bcl-2-like protein motifs are indicated by red boxes. (TIF) [file ppat.1013362.s004.tif]

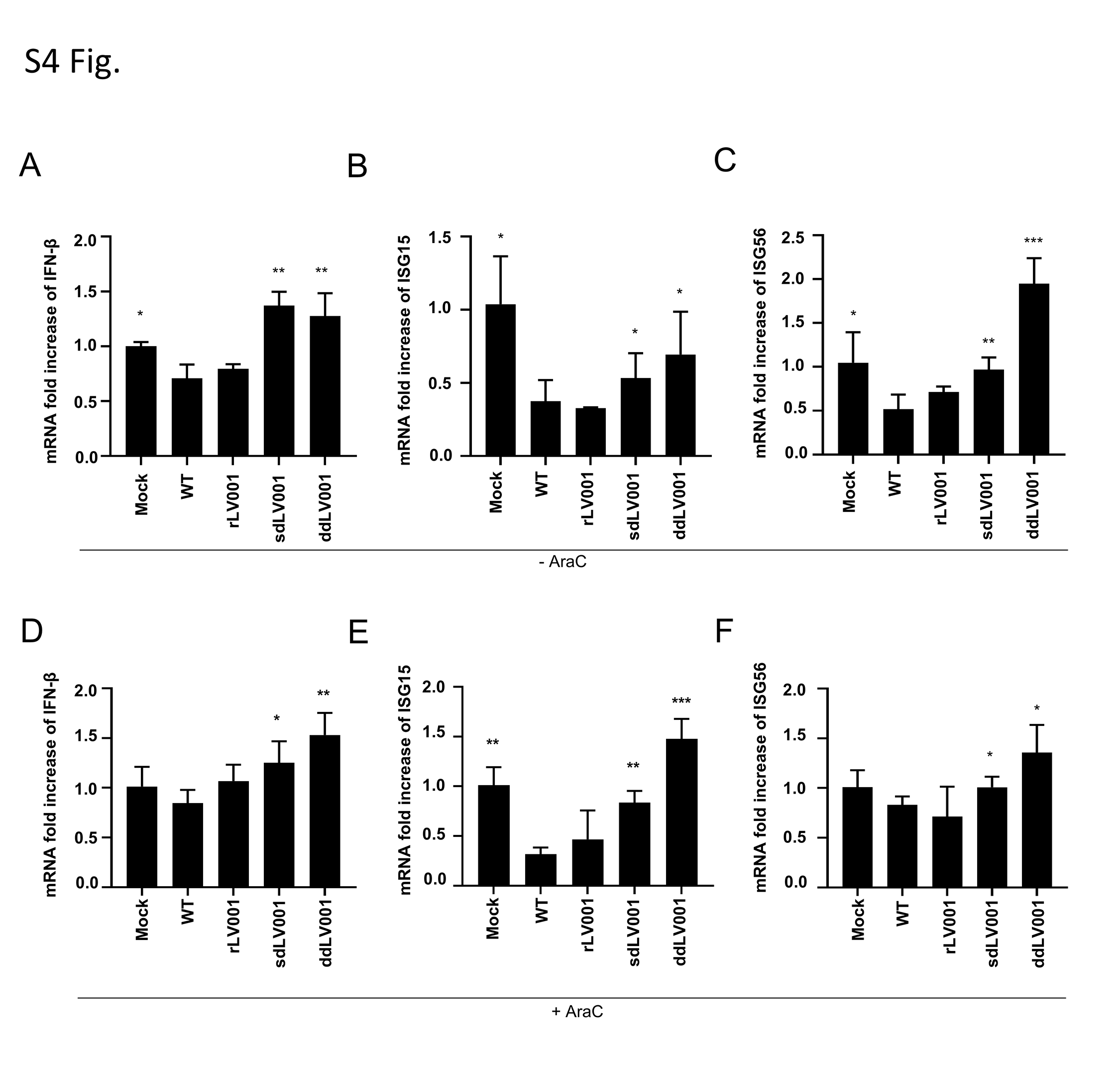

Supplement: S4 Fig — MDBK cells infected with LSDV-dd001/156, LSDV-sd001/156, LSDV r001-156-Myc revertant, or LSDV-WT (MOI = 5) were treated with (D–F) or left untreated (A–C) AraC. IFN-β, ISG15, and ISG56 mRNA levels were quantified by RT-qPCR at 24 hpi. (TIF) [file ppat.1013362.s005.tif]

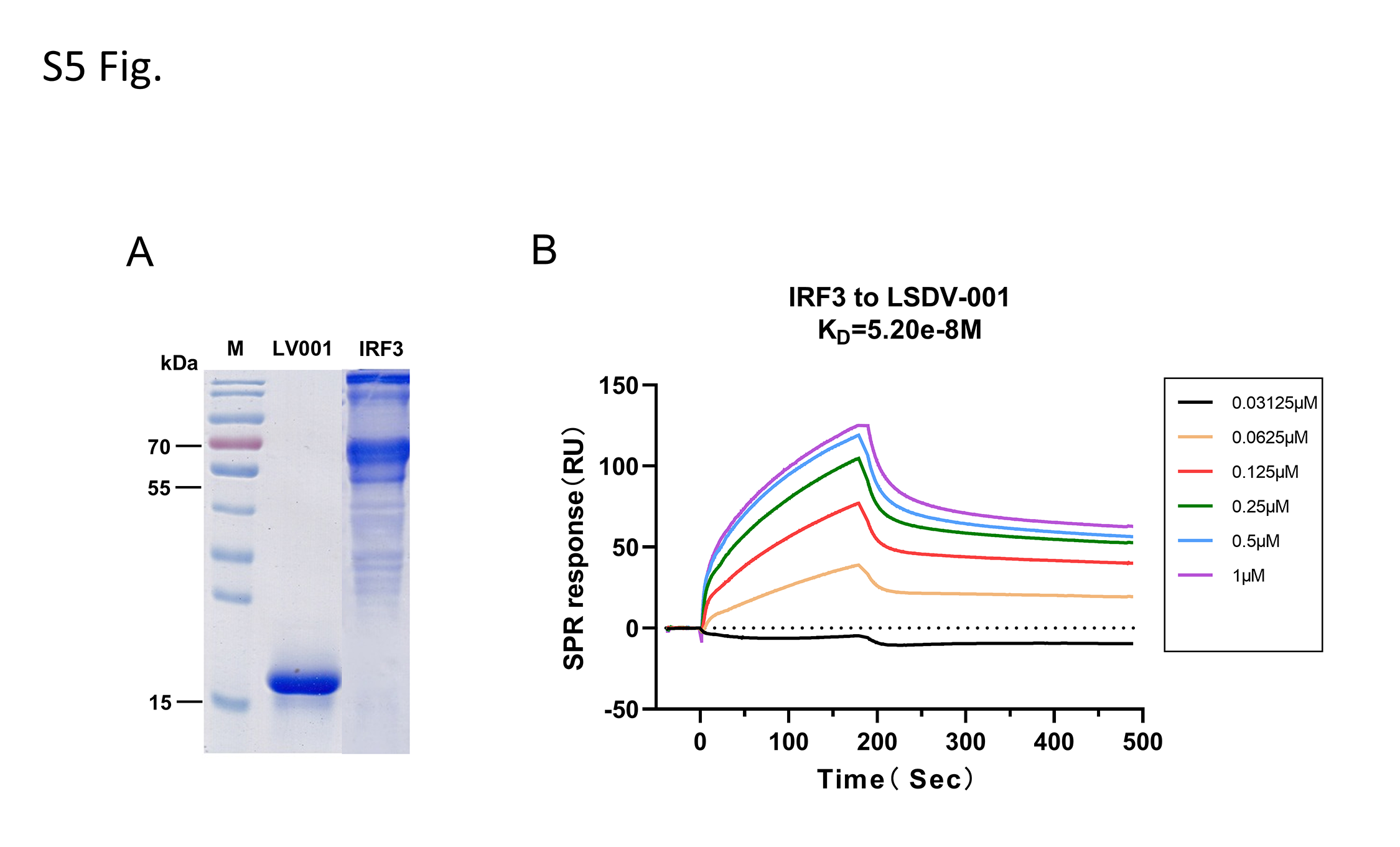

Supplement: S5 Fig — (A) Coomassie-stained SDS-PAGE of purified His-LV001 and GST-IRF3 expressed in E. coli. (B) SPR sensorgrams showing dose-dependent LV001 binding to immobilized IRF3 with calculated KD values. (TIF) [file ppat.1013362.s006.tif]

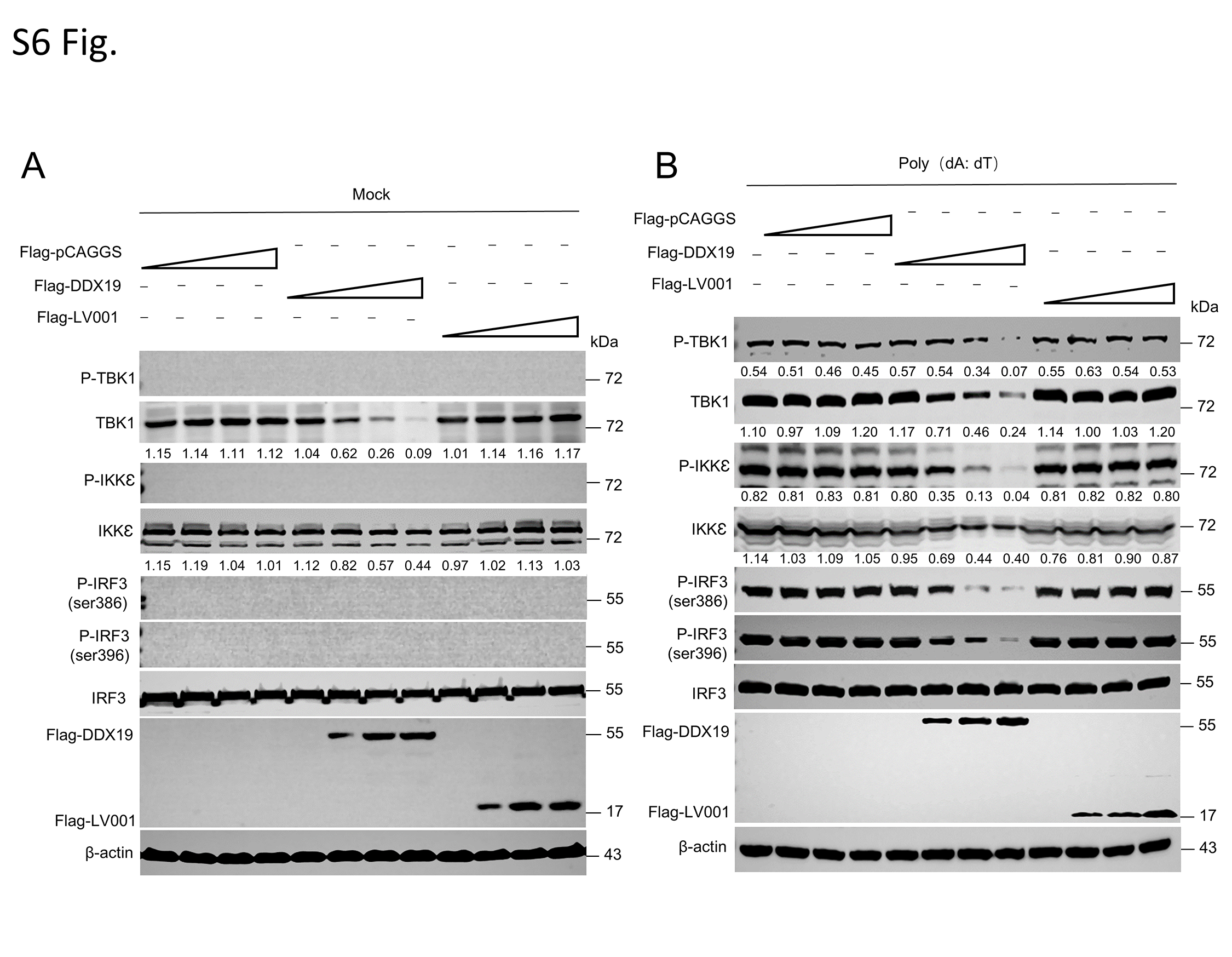

Supplement: S6 Fig — (A) Immunoblot analysis of HEK293T cells transfected with FLAG-LV001 or empty vector, probing for IRF3, p-IRF3 (Ser386/396), TBK1, p-TBK1, IKKε, and p-IKKε. (B) Same analysis as in (A) following poly(I:C) stimulation (12 h). β-actin served as a loading control. (TIF) [file ppat.1013362.s007.tif]

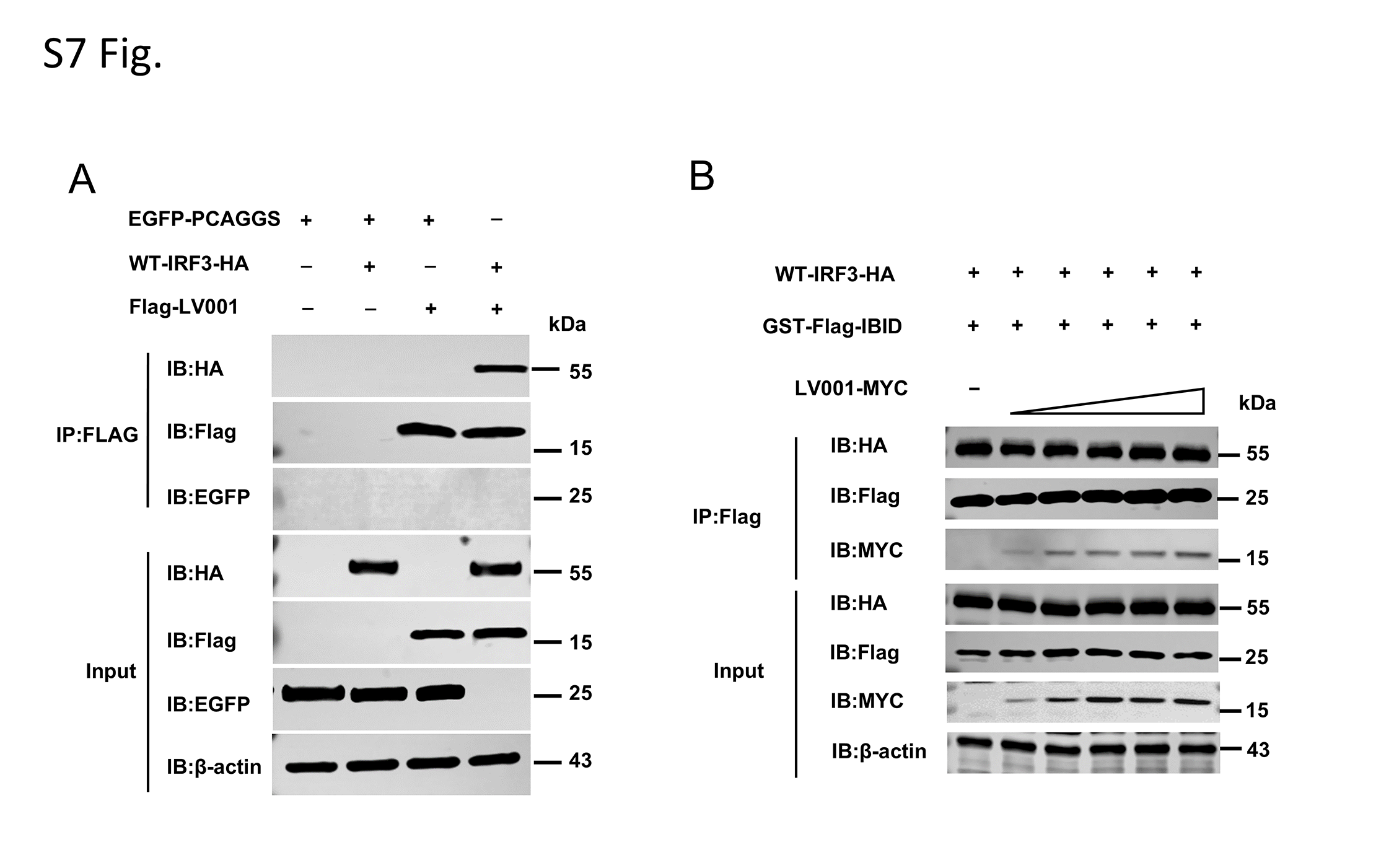

Supplement: S7 Fig — (A) Co-IP of HA-IRF3 and FLAG-p300/IBID in HEK293T cells expressing Myc-LV001 or EGFP control. (B) Dose-response co-IP with increasing Myc-LV001 (0.5-4 μg). Input lysates and IPs were analyzed by Western blot. (TIF) [file ppat.1013362.s008.tif]

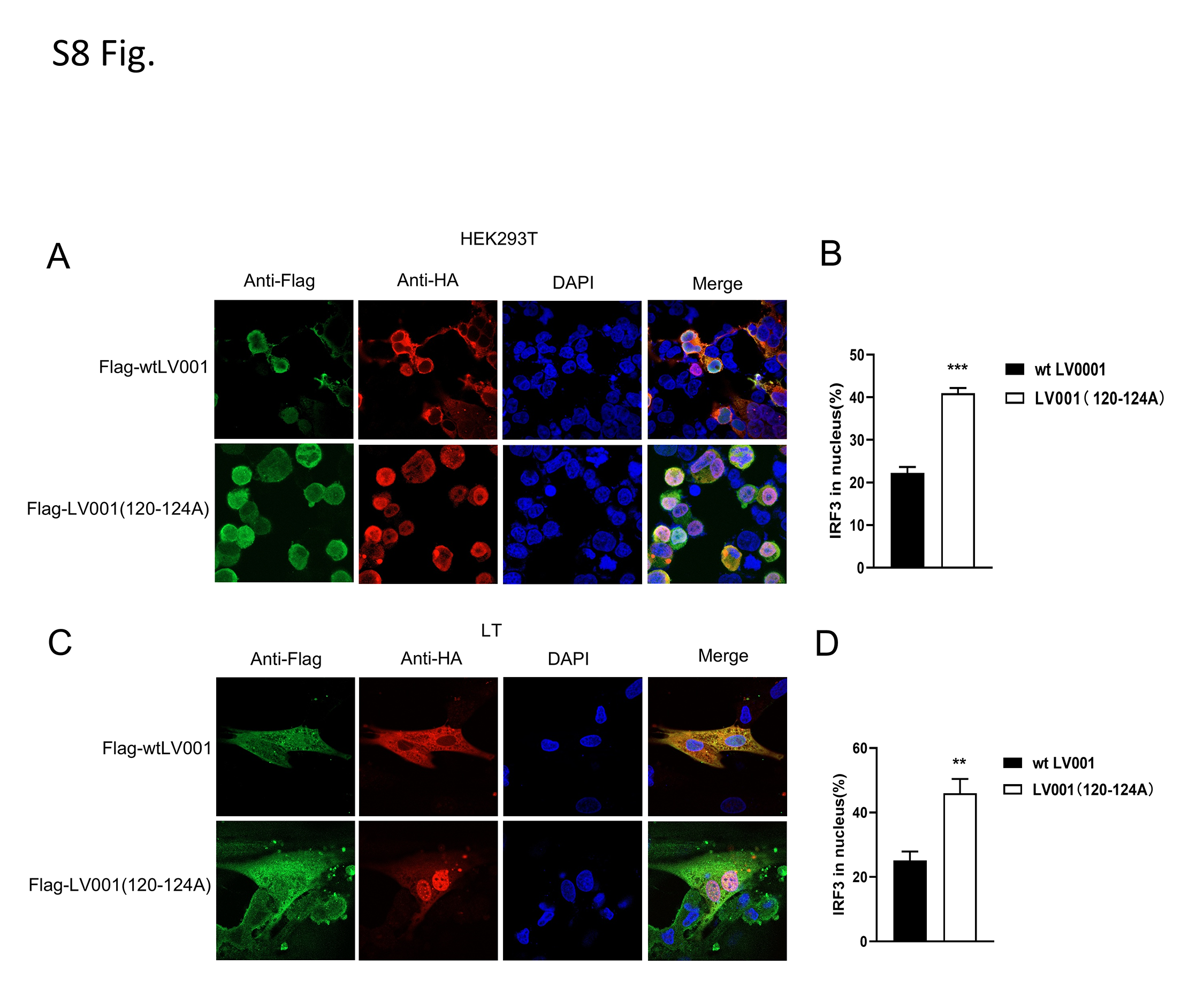

Supplement: S8 Fig — (A and C) Confocal microscopy of HEK293T/LT cells co-expressing IRF3/5D-HA with FLAG-LV001 or FLAG-LV001(120–124A). Nuclei (DAPI, blue), IRF3 (red), and LV001 (green) are shown. (B and D) Quantification of IRF3 nuclear translocation from ≥100 cells/condition. Data are represented mean ± SEM (*p < 0.05, **p < 0.01, ***p < 0.001 by Student’s t-test). (TIF) [file ppat.1013362.s009.tif]

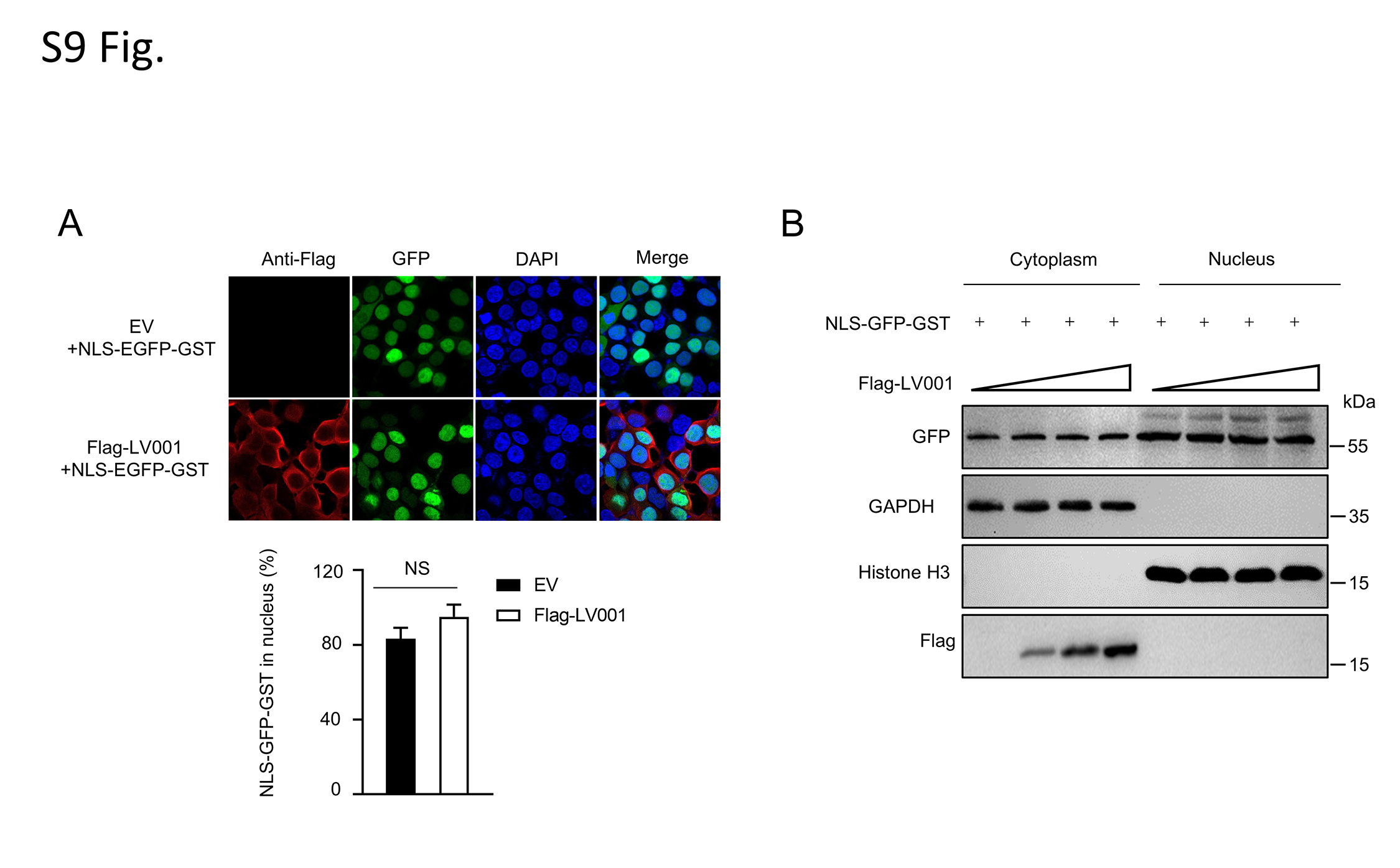

Supplement: S9 Fig — (A) HEK293T cells were transfected with FLAG-LV001 or an empty vector along with NLS-GFP-GST for 24 hpt. The subcellular localization of IRF3 (green), FLAG-LV001 (red), and cell nuclei (blue) was visualized using laser confocal microscopy. Nuclear translocation of NLS-GFP-GST was quantified from 100 cells per condition in different fields. Error bars denoted standard errors of the mean. (B) HEK293T cells were cotransfected with increasing amounts of FLAG-LV001 or an empty vector along with NLS-GFP-GST (1.5 μg). The distribution of NLS-GFP-GST in the nuclear and cytoplasmic compartments was detected by Western blotting. Histone H3 and GAPDH were used as markers for the nuclear and cytosolic compartments, respectively. (TIF) [file ppat.1013362.s010.tif]

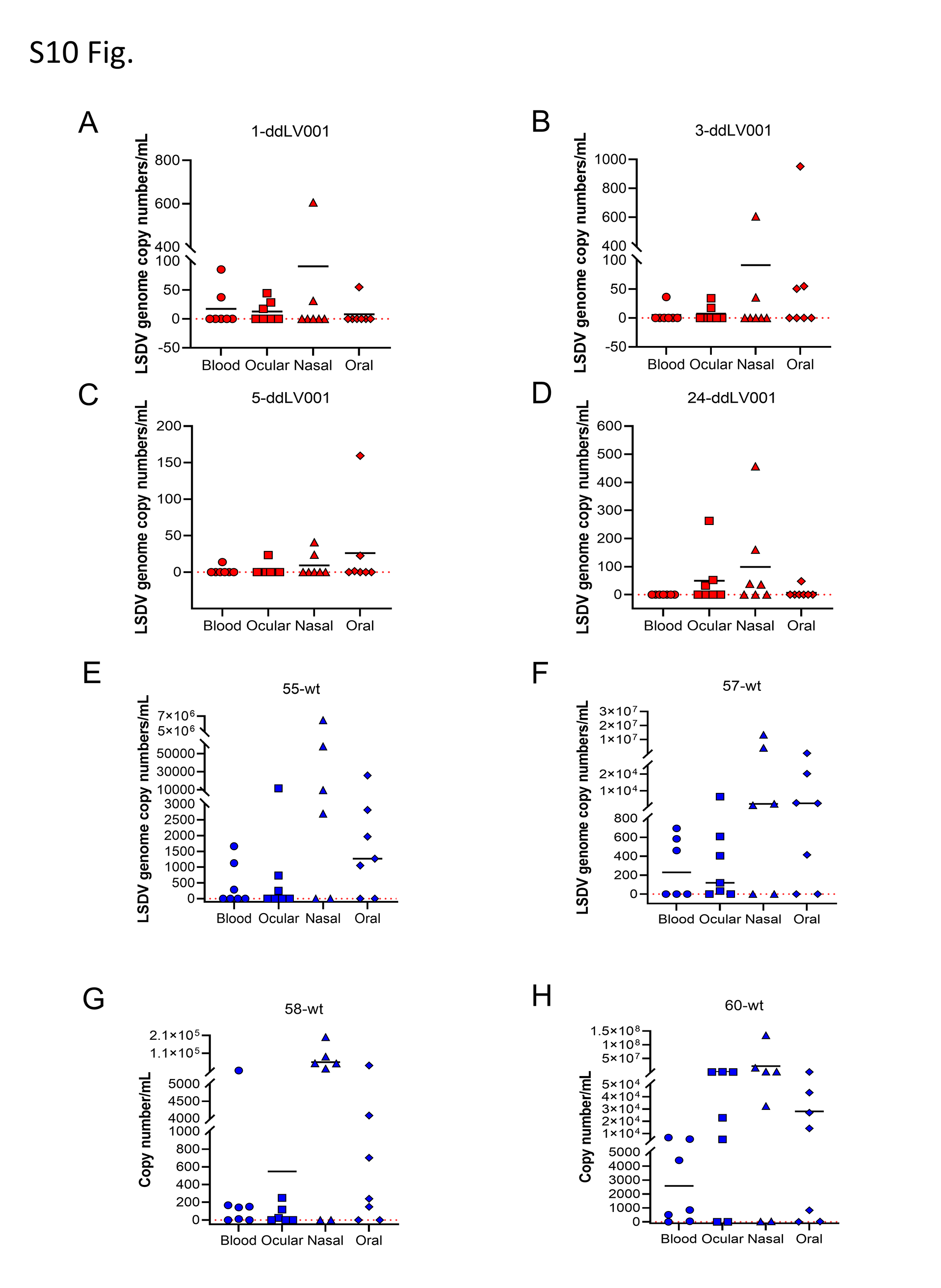

Supplement: S10 Fig — (A–D) Cattle infected with LSDV-dd001/156 exhibited detectable viral shedding at six different time points. (E–H) Cattle in the LSDV-WT group exhibited viral shedding at six corresponding time points. (TIF) [file ppat.1013362.s011.tif]

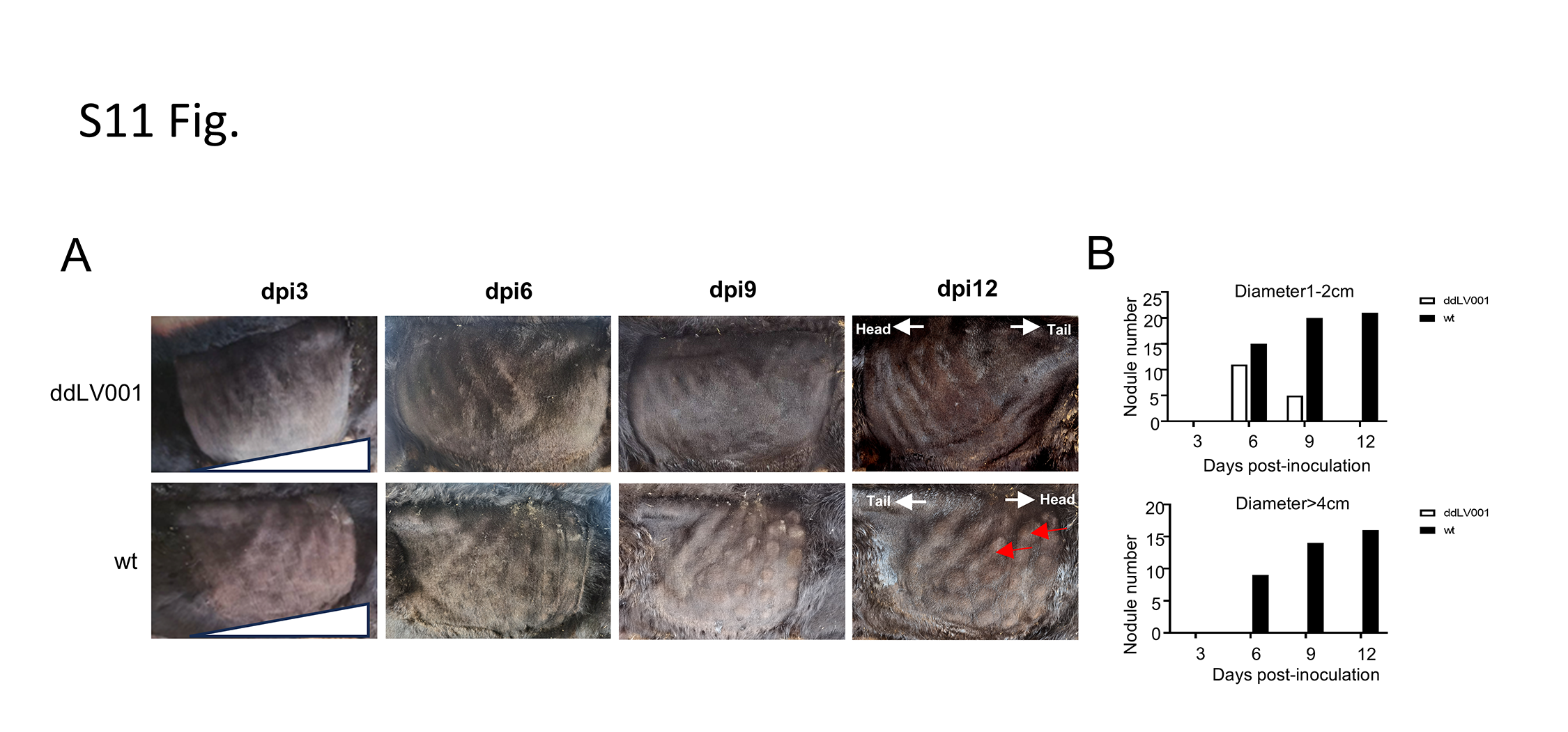

Supplement: S11 Fig — (A) Different doses of the two viruses were administered intradermally on opposite sides of the body to continuously monitor lesion formation. (B) Comparison of lesion counts following inoculation with the two viruses over the monitoring period. Photographs were taken by the authors during the animal experiments. (TIF) [file ppat.1013362.s012.tif]
